# Metabolic Reprogramming in Leaf Lettuce Grown Under Different Light Quality and Intensity Conditions Using Narrow-Band LEDs

**DOI:** 10.1038/s41598-018-25686-0

**Published:** 2018-05-21

**Authors:** Kazuyoshi Kitazaki, Atsushi Fukushima, Ryo Nakabayashi, Yozo Okazaki, Makoto Kobayashi, Tetsuya Mori, Tomoko Nishizawa, Sebastian Reyes-Chin-Wo, Richard W. Michelmore, Kazuki Saito, Kazuhiro Shoji, Miyako Kusano

**Affiliations:** 1 Central Research Institute of Electric Power Industry, Abiko, Chiba 270-1194 Japan; 2 RIKEN Center for Sustainable Resource Science, Yokohama, Kanagawa 230-0045 Japan; 3 UC Davis Genome Center, Davis, California 95616 USA; 4 Graduate School of Pharmaceutical Sciences, Chiba University, Chiba, Chiba 263-8522 Japan; 5 Graduate School of Life and Environmental Sciences, University of Tsukuba, Tsukuba, Ibaraki 305-8572 Japan; 6 Present Address: Research Faculty of Agriculture, Hokkaido University, Sapporo, Hokkaido 060-8589 Japan

## Abstract

Light-emitting diodes (LEDs) are an artificial light source used in closed-type plant factories and provide a promising solution for a year-round supply of green leafy vegetables, such as lettuce (*Lactuca sativa* L.). Obtaining high-quality seedlings using controlled irradiation from LEDs is critical, as the seedling health affects the growth and yield of leaf lettuce after transplantation. Because key molecular pathways underlying plant responses to a specific light quality and intensity remain poorly characterised, we used a multi-omics–based approach to evaluate the metabolic and transcriptional reprogramming of leaf lettuce seedlings grown under narrow-band LED lighting. Four types of monochromatic LEDs (one blue, two green and one red) and white fluorescent light (control) were used at low and high intensities (100 and 300 μmol·m^−2^·s^−1^, respectively). Multi-platform mass spectrometry-based metabolomics and RNA-Seq were used to determine changes in the metabolome and transcriptome of lettuce plants in response to different light qualities and intensities. Metabolic pathway analysis revealed distinct regulatory mechanisms involved in flavonoid and phenylpropanoid biosynthetic pathways under blue and green wavelengths. Taken together, these data suggest that the energy transmitted by green light is effective in creating a balance between biomass production and the production of secondary metabolites involved in plant defence.

## Introduction

A plant factory that can supply high-quality crops and vegetables all year-round holds a great promise to feed the growing world population in the face of future climate changes^1–3^. Closed-type plant factories can control the environmental conditions, of which light is one of the most important and easily controllable factors^4^. Light-emitting diodes (LEDs) are currently the most commonly used artificial light sources in plant factories. Vegetables (radish^5^ and buckwheat^6,7^), fruits (strawberry^8^ and grapes^9^) and leafy greens (spinach^10,11^ and lettuce) have been grown in controlled environments to maximise their yield and nutritional content. Leafy greens grown in a closed-type plant factory are served directly without washing, as no pesticides are used in their cultivation. Among the leafy greens, lettuce (*Lactuca sativa* L.) is of high commercial and nutritional value because of the high accumulation of anthocyanin, a type of flavonoid, which imparts the characteristic red colour to the leaves of red leaf lettuce. Moreover, flavonoids possess a high antioxidant activity and have potential beneficial effects on human health^12,13^.

Light is not only an essential energy source for plant growth but also plays a critical role in plant morphogenesis and physiology, such as hypocotyl elongation, leaf area expansion and metabolism via light signalling response^14,15^. Plants are usually highly sensitive to the type and intensity of light during initial growth stages^15^. To determine the effect of light quality on plant growth, studies have been conducted using blue light, red light and a combination of the two^11,16–19^. Both blue and red lights are major energy sources for photosynthesis and are recognised by photoreceptors, i.e., cryptochrome, phototropin and phytochromes that regulate plant growth. Cryptochrome and phototropin monitor specific blue light wavelengths, whereas phytochromes are capable of detecting nanometre-level differences in red light wavelengths approximating 700 nm. Each photoreceptor elicits a specific photomorphogenic response to light^7,20^. Additionally, several studies have highlighted the physiological role of green light (495–570 nm) in plants, including stomatal conductance and early stem elongation^21–24^, although the specific role of green light in plant growth and development remains unclear. To determine the optimal light conditions using LED technology for vegetable production in plant factories, it is critical to understand the effect of monochromatic light at narrow bandwidth on plant growth and development at a molecular level. However, global metabolic and transcriptional responses to monochromatic light delivered by LEDs of different colours remain under-characterised and poorly understood. It is also unclear whether small changes in the light spectrum of green light elicit detectable effects in plant growth and development.

High-throughput technologies, genomic sequencing, RNA-Seq and metabolite profiling using mass spectrometry (MS) facilitate the systematic monitoring of the cellular responses in the genome, transcriptome, proteome and metabolome (e.g., see^25,26^). RNA-Seq has been used to assess the transcriptomic changes in lettuce in response to biotic stress^27^, to identify anthocyanin biosynthesis genes in red leaf lettuce^28,29^ and to investigate the seed transcriptome in lettuce plants^30^. Higashi and co-workers previously used RNA-Seq to identify common oscillating transcripts in lettuce plants^31^. The metabolomic approach has been used to characterise changes in lettuce metabolome in response to zinc and salt stress^32^, oxidative stress^33^ and UV-B irradiation^34^ and to determine the synergistic effects of monochromic LED combined with high CO_2_ and nutrients^35^. A systems biology approach with multiple omics level studies facilitates not only the production of cellular component inventories but also the identification of key pathways and regulatory sites of complex plant responses to LEDs with different wavelengths.

In this study, we used systems biology approach to evaluate the impact of variable light quality and intensity using narrow-band LED lighting on metabolic and transcriptional reprogramming in leaf lettuce. Four types of narrow-band monochromatic LEDs were used, including a blue LED panel, two green LED panels and a red LED panel. Different photosynthetic photon flux densities (PPFDs) were applied, including 100 and 300 μmol·m^−2^·s^−1^ (hereafter referred to as P100 and P300, respectively). MS-based metabolomics and Illumina-based RNA-Seq were used to capture changes in the metabolome and transcriptome, respectively, in response to variable light quality and intensity. Data obtained in this study highlight key potential metabolic pathways and morphological variations in leaf lettuce.

## Results

### Experimental Setup

**Figure 1:**
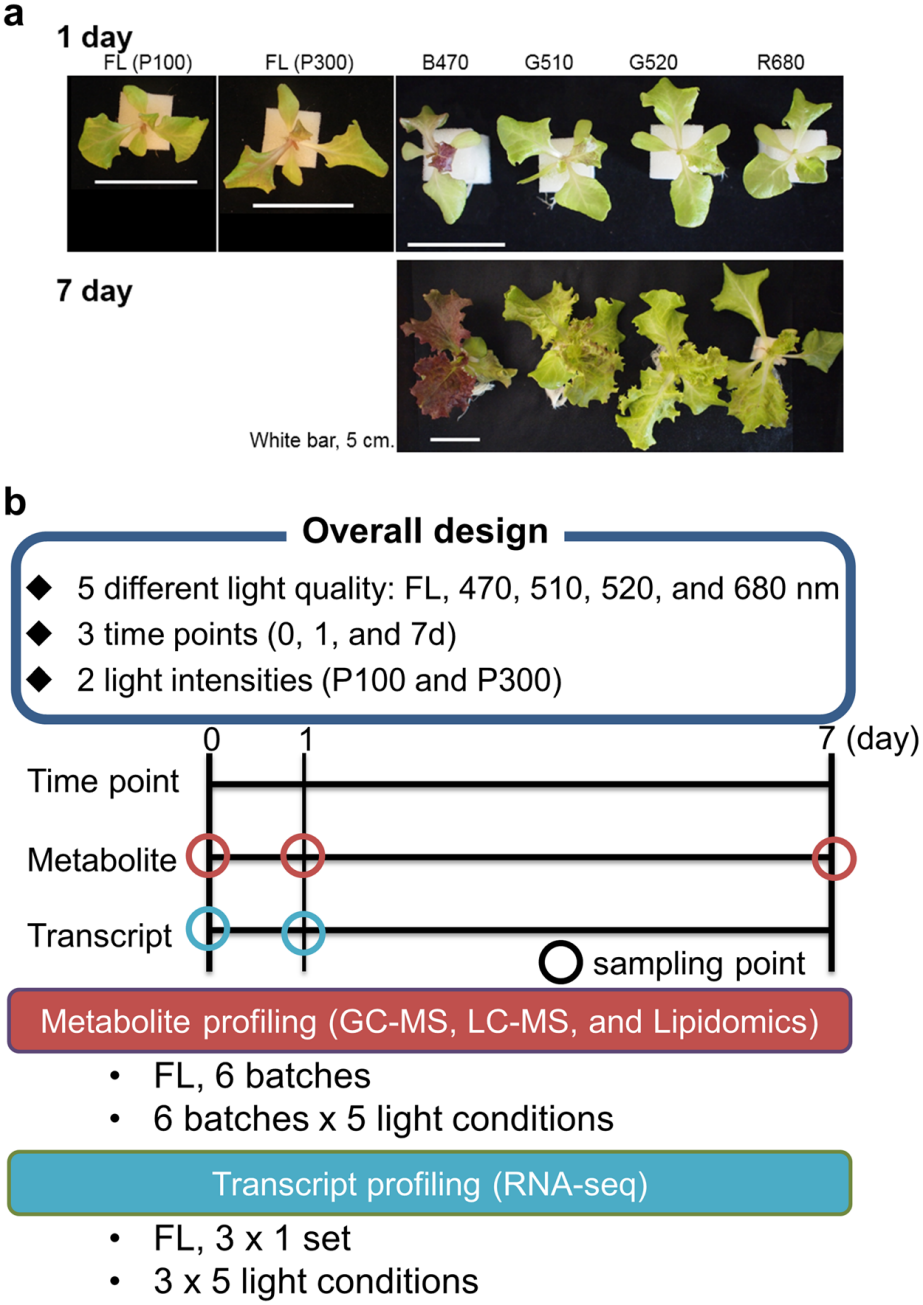
Overview and experimental setup used in this study. (**a**) Representative leaf morphology of red leaf lettuce plants treated with different light qualities (days 1 and 7 at P300). (**b**) Overall design of this study. A lamp emitting white light (FL; control) and four different light-emitting diodes (LEDs) [blue, 470 nm (B470); green, 510 nm (G510) and 524 nm (G520); and red, 680 nm (R680)] at two light intensities (P100 and P300). Data were recorded on 0, 1 and 7 days after sowing, and plants were photographed 17 days after sowing. Scale bars indicate 5 cm. The total photosynthetic photon flux density (PPFD) was 100 and 300 µmol·m^−2^·s^−1^.

To reveal the molecular mechanisms of light signalling, we first analysed changes in the metabolome of lettuce in response to change in light intensity and quality. As a proof-of-concept analysis, we evaluated the metabolite profiles of leaf lettuce exposed to blue and red LEDs, based on previously established conditions using broad-band LEDs^16^. Our multivariate statistical analyses showed that samples clustered in different areas of the scatter plot, with each cluster representing plants exposed to red LEDs, blue LEDs and white fluorescent light (Figs S1a and b). The score scatter plot showed that variation between the different light qualities was more pronounced along the first principal component axis (PC1), which explained the highest variation in the models. To determine the metabolites responsible for differences in samples exposed to red and blue LEDs, loading plots were constructed, which identified amino acids, lipids and flavonoids as potential contributors (Fig. S1c). Based on the result of these experiments, we decided to setup the overall experimental design using four narrow-band LEDs, including a blue LED panel, two green LED panels and a red LED panel. The peak wavelengths were 470 nm for the blue panel (B470), 510 and 524 nm for green panels (G510 and G520, respectively) and 680 nm for the red panel (R680). White fluorescent light (FL) was used as control. The effect of different light sources on the growth and development of lettuce was monitored at three time points (days 0, 1 and 7) and two light intensities (P100 and P300) (Fig. 1). The third leaf of lettuce plants was used to detect metabolites and intermediates in central metabolism and secondary metabolites, such as anthocyanins and lipids^36–39^ using gas and liquid chromatography followed by mass spectrometry (GC–MS and LC–MS, respectively). Additionally, Illumina-based RNA-Seq analysis was performed to assess the effect of different light treatments on lettuce transcriptome. This parallel assessment of metabolomic and transcriptomic changes would provide valuable insights into the regulatory mechanisms underlying the physiological responses of lettuce plants to different light qualities and intensities.

### Light-Mediated Perturbations in Metabolite Levels of Lettuce Plants

#### Overview of metabolites

**Figure 2:**
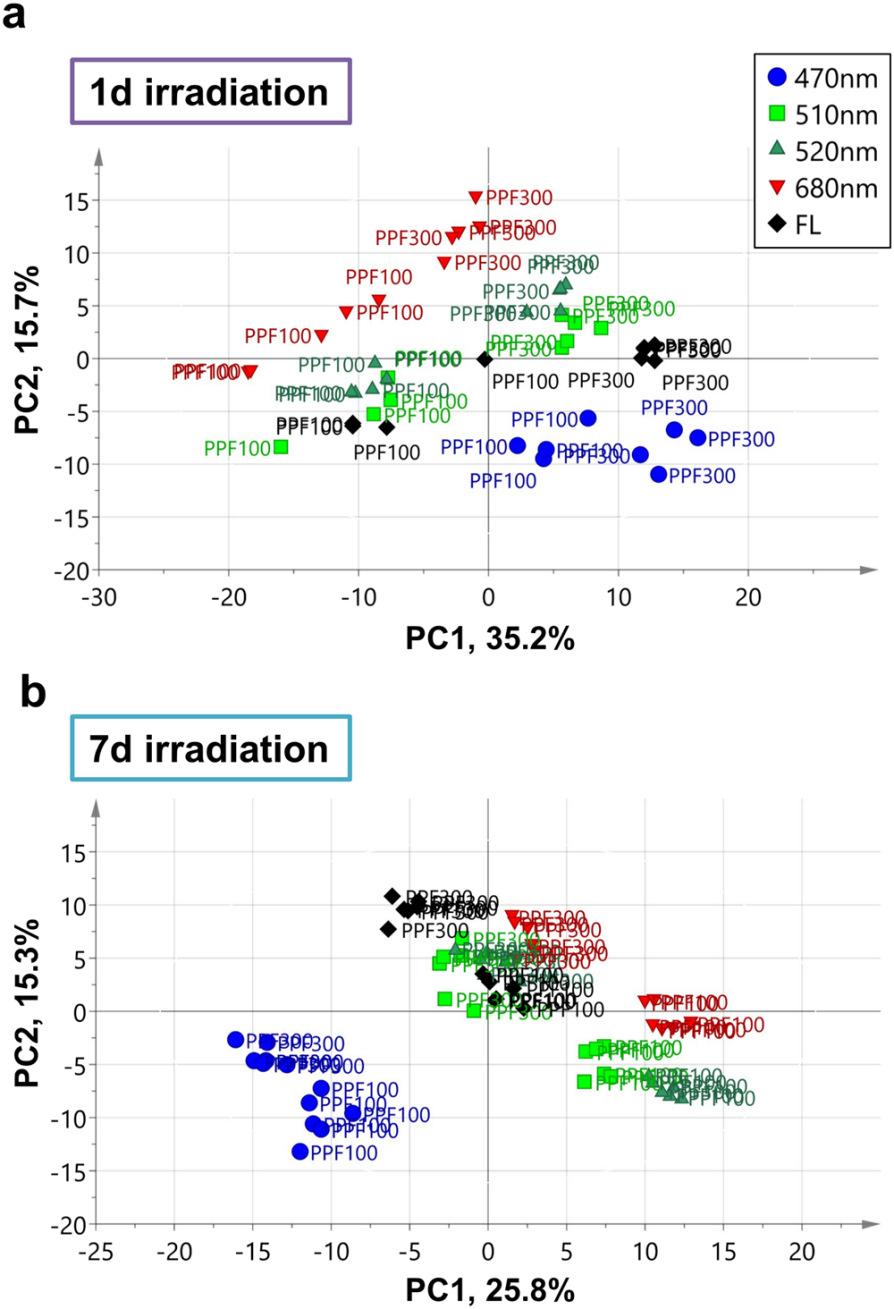
O2PLS-DA score scatter plots showing distinct metabolite profiles of lettuce plants grown under different light qualities and intensities. Metabolic responses of lettuce leaves subjected to (**a**) short (1-day) and (**b**) prolonged (7-day) exposure to different light qualities [FL = white fluorescent light; B470 = blue (470 nm); G510 and G520 = green (510 and 524 nm, respectively); R680 = red light (680 nm)] and light intensities (P100 and P300). Each symbol indicates an independent plant sample in the score scatter plot (biological replicates, *n* = 6). The *p*-value was calculated by ANOVA of cross-validated residuals (CV-ANOVA); the significance level was set at *p* < 0.01 for the model.

Using multi-MS-based metabolomics^40^, we detected 2,357 peaks, including 275 identified/annotated metabolites, in lettuce leaves in response to a combination of different light qualities and intensities (Supplementary Table S1 and Fig. S2). Of these 275 metabolites, 127 were involved in central metabolism, 110 were lipids and 38 were polar secondary metabolites detected using GC–MS and LC–MS, respectively. These include tentatively identified/annotated polar secondary metabolites, lettuce-specific sesquiterpenes, such as lactucin and lactucopicrin. To visualise and inspect the sample distribution and changes in metabolite levels under different light qualities, we performed orthogonal partial least square discriminant analysis (O2PLS-DA), a supervised multivariate statistical method (Fig. 2). The score scatter plot showed a clear separation between lettuce plants exposed to long- and short-term irradiation, indicating distinct metabolite compositions of these plants in response to different light qualities and intensities (Figs 2 and S3).

We also assessed changes in the content of primary and secondary metabolites in leaf samples of lettuce exposed to different light qualities (Fig. S2). Complex abundance patterns were observed in the case of sugar metabolism and tricarboxylic acid (TCA) cycle intermediates. For example, sucrose increased in response to red light, whereas trehalose was largely unchanged (Fig. S2a). The accumulation of 2-oxoglutarate (2OG) was higher under R680 than under FL and B470 (Fig. S2b). The level of glutamine (Gln), glutamic acid (Glu) and aspartic acid (Asp) was lower after 7 days of irradiation than after 1 day, irrespective of the wavelength of light (Fig. S2c). Stress-related metabolites exhibited more complex profiles (Fig. S2d). Metabolite accumulation patterns of branched chain and aromatic amino acids were similar to those of Gln, Glu and Asp (Fig. S2e). Among the secondary metabolites, major chlorogenates, such as caffeoyl quinate, dicaffeoyl quinate and quercetin derivatives, were induced by B470 irradiation (Fig. S2g).

#### Identification of differentially accumulated metabolites

**Figure 3:**
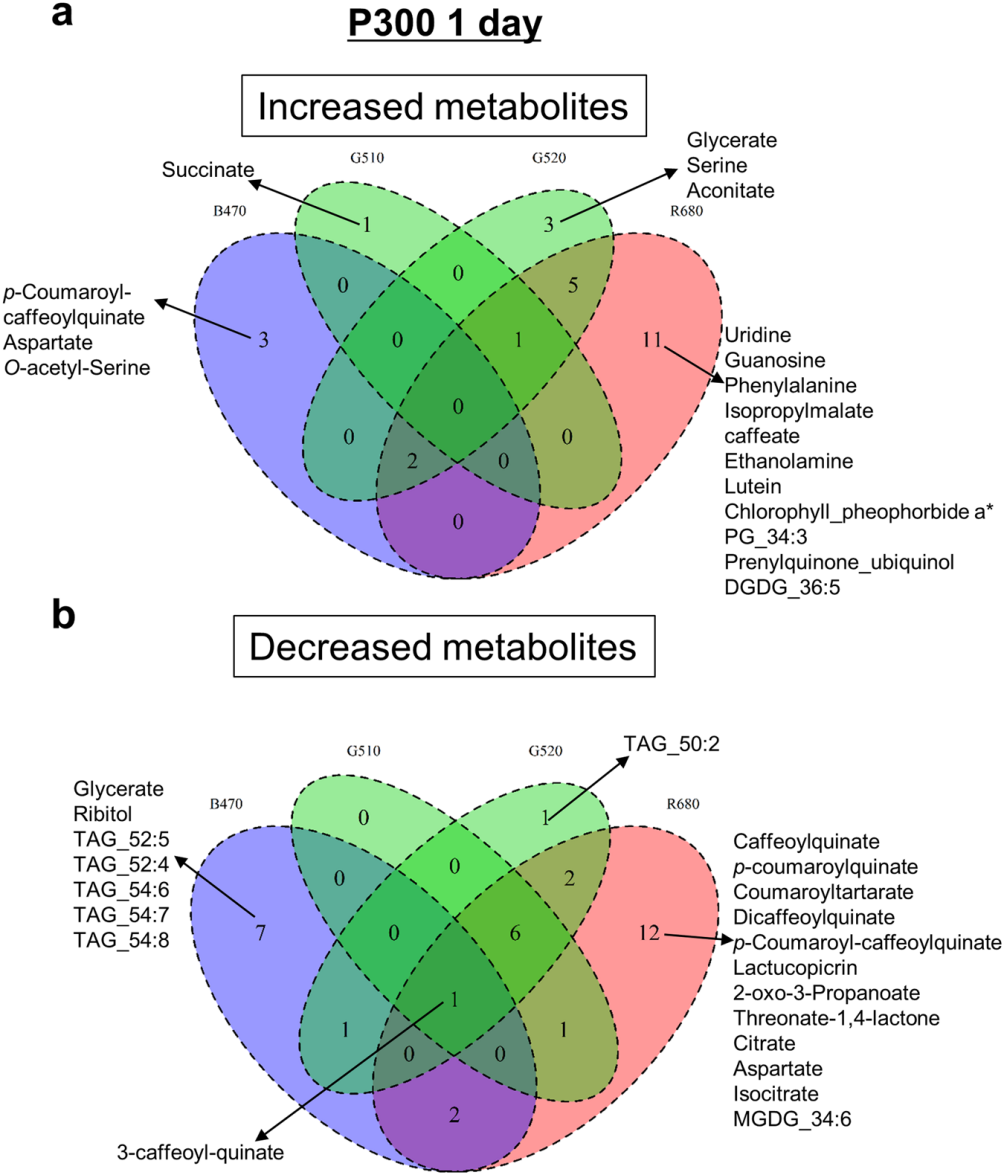
Venn diagrams showing differentially accumulated metabolites. We analysed differentially accumulated metabolites from the lettuce samples exposed to white fluorescent light (FL) or LEDs with different wavelengths and intensities. Statistically significant changes in metabolite levels compared with FL exposure were identified using LIMMA^71^. (**a**) increased metabolites and (**b**) decreased metabolites after 1 day of irradiation are shown. The significance level was set as |log_2_ fold-change| ≥ 1, FDR < 0.05.

To identify light-responsive metabolites, we identified metabolites whose accumulation was either increased or decreased post-irradiation with blue, green and red LEDs compared with FL control (|log_2_ fold-change| ≥ 1 and false discovery rate, FDR < 0.05). Venn diagrams (Fig. 3 and Supplementary Fig. S4) were constructed to summarise the number of metabolites that differentially accumulated in response to different light qualities at P300 (1 and 7 day-treatment) relative to FL and the overlap between each set of metabolites. Changes in the level of some metabolites were common to all LED sources, whereas others were specific to a particular light source in comparison to FL. For example, the level of 3-caffeoyl-quinate was reduced in plants exposed to all types of LEDs compared with those exposed to FL (Fig. 3b). By contrast, levels of Asp, *O*-acetyl-serine and *p*-coumaroyl-caffeoylquinate were increased in plants exposed to B470, whereas glycerate, serine (Ser) and *cis*-aconitate were increased in those exposed to G520 after one day. A total of 11 metabolites were increased in plants irradiated with R680 for one day compared with those exposed to FL.

The number of light-responsive metabolites was much higher after 7 days of irradiation than after one day. Plants exposed to B470 for 7 days showed increased levels of amino acids, fatty acids, lipids and alpha-tocopherol (vitamin E) compared with those exposed to FL for 7 days (Fig. S4a). In comparison to a 7-day exposure to FL, a 7-day exposure to green light increased the levels of succinate, 15-deoxylactucin-8-sulphate, lactucopicrin-15-oxalate, beta-sitosterol and sulphoquinovosyldiacylglycerol (SQDG) 34:1, whereas the same duration of exposure to R680 increased the levels of caftarate, isopropylmalate, glycolate, homoserine, tartrate and ubiquinol. Additionally, a number of metabolites were decreased following LED exposure compared with FL treatment; G510 irradiation decreased amino and fatty acid levels, G520 exposure decreased lanosteryl linolenate, 24-methylenecycloartanyl linoleate and 24-methylenecycloartanyl linolenate levels and B470 decreased the levels of sugars, caffeate derivatives and four types of lipids, including phosphatidylglycerols (PGs), monogalactosyldiacylglycerols (MGDGs), triacylglycerols (TAGs) and digalactosyldiacylglycerols (DGDGs) (Fig. S4b).

### Genome-Wide Transcriptome Changes in Response to Different Light Qualities and Intensities

#### Identification of differentially expressed genes and pathway analysis

For a deeper understanding of the metabolic responses to different light qualities and intensities, RNA-Seq was performed (Fig. S5 and Supplementary Table S2), and statistical analysis was conducted to identify differentially expressed genes (DEGs) under different light qualities in comparison to treatment. Venn diagrams (Fig. S6) show DEGs that overlapped among samples irradiated with different colours and intensities of LEDs.

**Figure 4:**
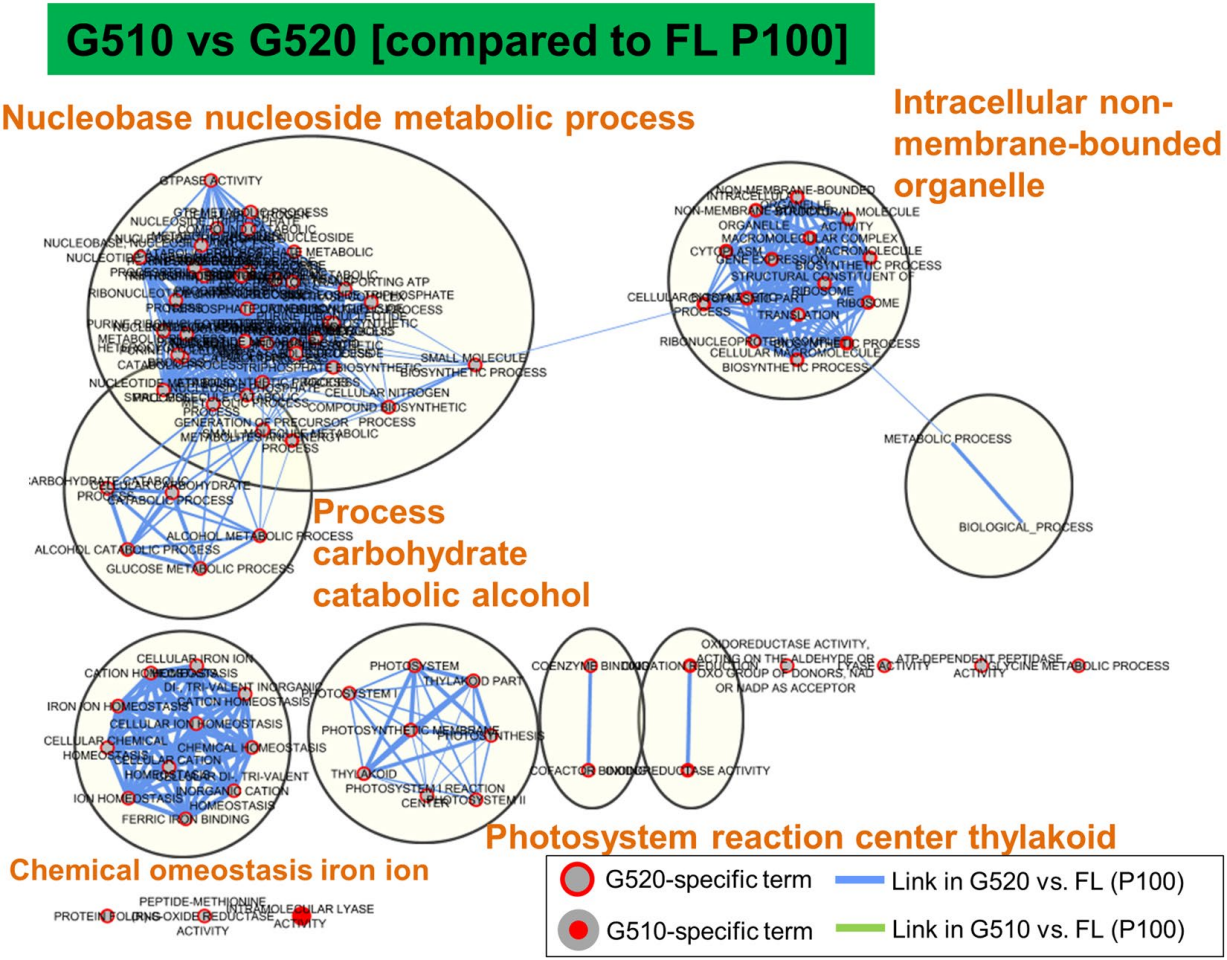
Functional enrichment analysis of transcriptome data. The enrichment map (EM) shows biological processes occurring under G510 and G520 light. Hypergeometric tests of gene ontology (GO) terms were performed using BiNGO software^69^. The map shows enriched GO terms related to differences in transcriptome between G510 and G520. Comparison is against FL. Nodes indicate the GO terms and edges between nodes; the gene overlap in GO terms is shown. The inner circle size of each node indicates the number of differentially expressed genes (DEGs) in ‘comparison 1’ (G510 vs. FL) within the GO term in the biological process. The node border size represents the number of DEGs in and ‘comparison 2’ (G520 vs. FL) within the GO term in the biological process. The colour of the node and its border shows the significance based on the BiNGO FDR of the GO term for ‘comparison 1’ and ‘comparison 2’, respectively. Solid red nodes indicate major GO functional terms. The edge size represents the number of DEGs that overlap between two linked GO terms (Jaccard coefficient, cutoff = 0.25). Sole green edges correspond to both data sets when it is the only colour edge. Green and blue edges represent ‘comparison 1’ and ‘comparison 2,’ respectively. Blue dotted circles show summarised GO term clusters based on AutoAnnotate^70^. Enrichment maps were created using the Cytoscape (v3.2.1) Enrichment Map plugin^41,42^.

To assess gene expression patterns in response to irradiation, we investigated the gene ontology (GO) enrichment of DEGs and then used Enrichment Map^41,42^ based on hypergeometric tests of GO terms. The significantly enriched GO terms obtained with G510 versus G520 irradiation (P100) were shown (Fig. 4). The five largest GO clusters obtained in G520-specific data set were ‘nucleobase nucleoside metabolic process’, ‘intracellular non-membrane-bound organelle’, ‘process carbohydrate catabolic alcohol’, ‘chemical homeostasis iron ion’ and ‘photosystem reaction centre thylakoid’. Enrichment maps in Figs S7–S9 show biological processes enriched under different light qualities. Three significantly enriched GO term clusters were identified as B470-specific terms from transcript profiles associated with B470 and G510 irradiation (P100), including ‘intracellular organelle cellular processes’, ‘photosystem reaction centre’ and ‘catabolic process amino acid’ (Fig. S7). Several GO term clusters were identified from B470 versus G520 comparison (P100) (Fig. S8). Among these, three GO term clusters were G520-specific, including ‘purine ribonucleotide process’, ‘small carbohydrate glucose process’ and ‘chemical homeostasis’. Comparison between R680 and G520 responses (P300) revealed ‘catabolic process’ and ‘polyol metabolic process’ as significant GO clusters (Fig. S9). Among these, only the GO cluster ‘carbon-carbon lyase’ was G520-specific. These findings suggest an essential discrepancy in transcript levels between G510 and G520 irradiation.

To thoroughly investigate gene expression patterns in the primary and secondary metabolism of lettuce, we performed MAPMAN analysis^43^. For metabolism, remarkable changes were noted for expression patterns of many genes either directly or indirectly involved in secondary metabolism (Fig. S10). Genes encoding enzyme candidates putatively involved in flavonoid biosynthesis, including chalcone synthase (CHS), chalcone-flavanone isomerase (CHI) and flavonol synthase (FLS) showed coordinated reduction in transcript levels in response to green light (G510 and G520; P300).

#### Validation of the RNA-Seq results using quantitative reverse transcription PCR

**Figure 5:**
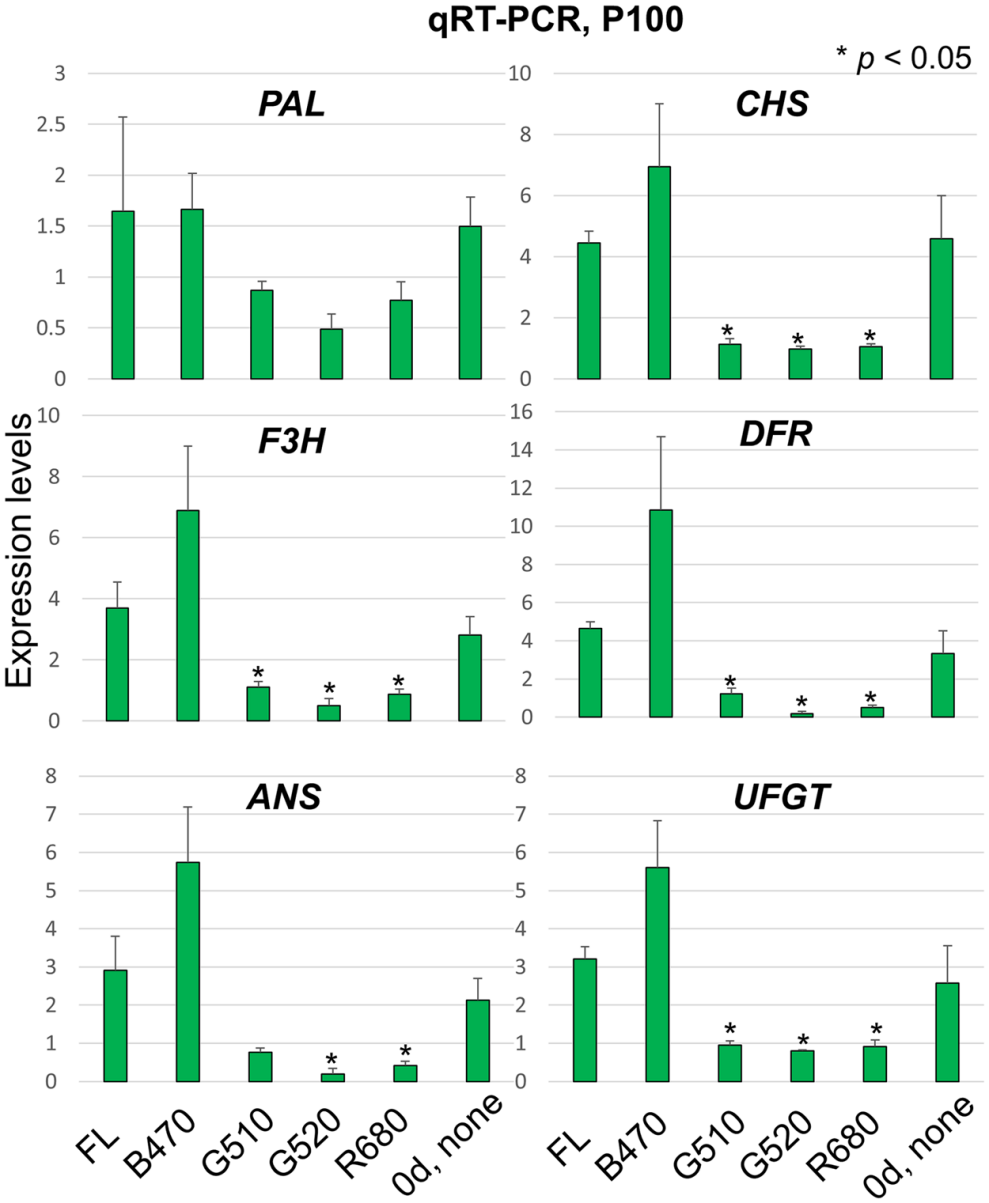
Validation of RNA-Seq data using qRT-PCR. Red leaf lettuce plants were exposed to different light qualities at P100 [FL, white fluorescent light; B470, blue (470 nm); G510 and G520, green (510 and 524 nm, respectively); R680, red light (680 nm)] for 24 h. Expression levels of six genes involved in flavonoid biosynthesis were subjected to qRT-PCR; they were *phenylalanine ammonia-lyase* (*PAL*), *chalcone synthase* (*CHS*), *flavanone 3-hydroxylase* (*F3H*), *dihydroflavonol 4-reductase* (*DFR*), *anthocyanidin synthase* (*ANS*) and *UDP-glucose:flavonoid 3-*O*-glucosyltransferase* (*UFGT*). Three biological replicates were performed for each gene. Welch’s *t*-test was used to calculate the *p*-values. Statistically significant differences in expression levels are indicated using an asterisk (*p* < 0.05). Gene-specific primers used for qRT-PCR are listed in Supplementary Table S3. d, day.

To validate RNA-Seq data, we examined six flavonoid biosynthesis genes that were significantly down-regulated by green and red LEDs, i.e. *phenylalanine ammonia-lyase* (*PAL*), *CHS*, *flavonoid 3-hydroxylase* (*F3H*), *dihydroflavonol 4-reductase* (*DFR*), *anthocyanidin synthase* (*ANS*) and *UDP-glucose:flavonoid 3-*O*-glucosyltransferase* (*UFGT*) using quantitative reverse transcription PCR (qRT-PCR) (Fig. 5). qRT-PCR analysis confirmed that the expression of these genes was reduced after exposure to green and red LEDs to levels lower than those achieved after FL exposure (Fig. S11). Moreover, B470 treatment up-regulated gene expression, irrespective of the light intensity (Fig. S11).

### Integrated Omics Analysis Revealed the Distinct Regulatory Mechanisms Involved in the Biosynthetic Pathways of Flavonoids and Chlorogenates

**Figure 6:**
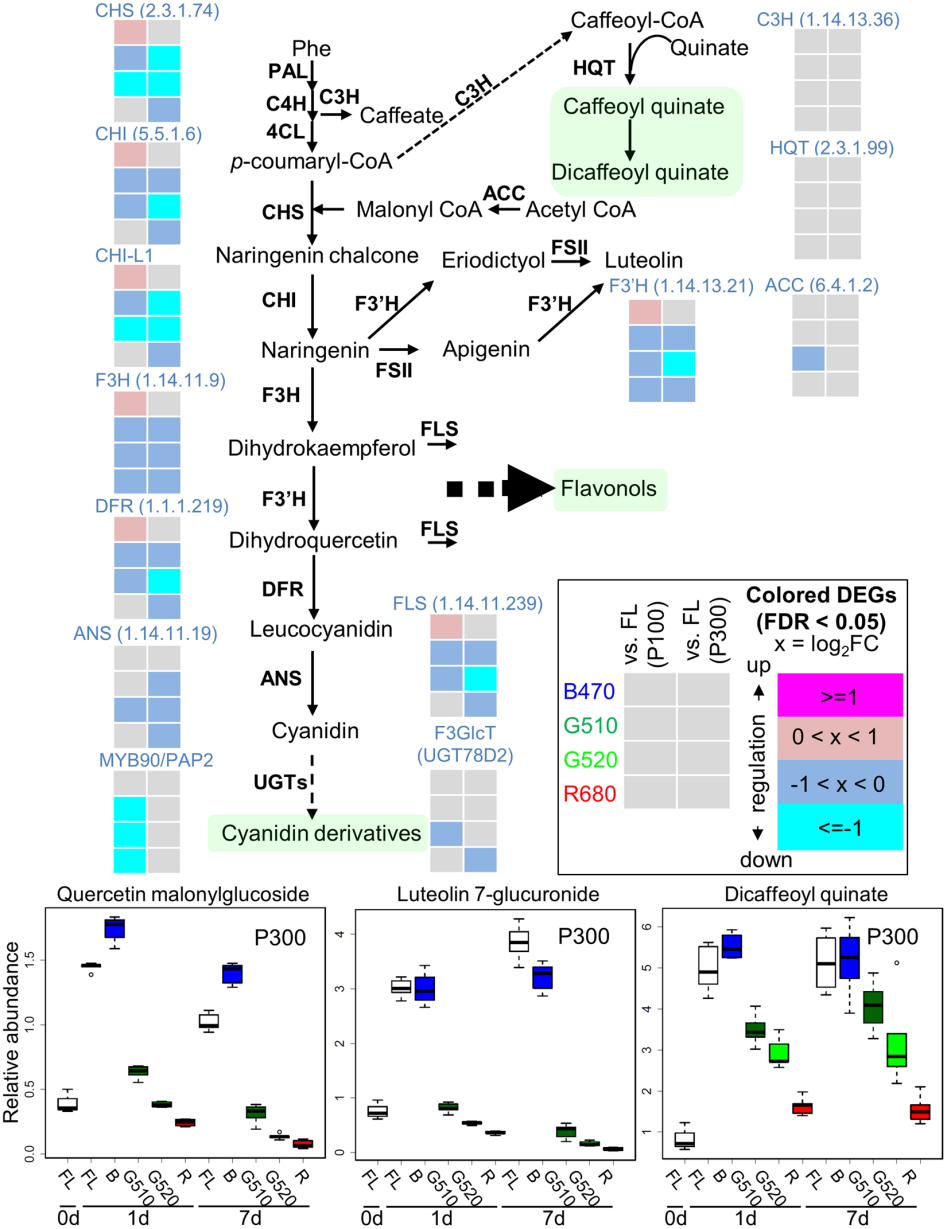
Integrated pathway analysis of metabolome and transcriptome data obtained under different light qualities. The biosynthetic pathways and expression profiles of genes involved in the biosynthesis of phenylpropanoid and flavonoid skeletons are displayed. Fold changes (e.g., G510 vs. FL) in expression levels of genes encoding enzymes involved in the phenylpropanoid biosynthetic pathway are shown. ACC, acetyl-CoA carboxylase; C4H, cinnamate 4‐hydroxylase; 4CL, 4‐hydroxycinnamoyl CoA ligase; F3′H, flavonoid 3′-hydroxylase; FSII, flavone synthase II; FLS, flavonol synthase; F3GlcT, UGT78D2; C3H, Coumarate-3-hydroxylase; HQT, hydroxycinnamoyl CoA quinate transferase; and FL, white fluorescent light.

Lastly, we used an integrated systems biology approach to identify pathways that were altered in lettuce plants exposed to different light qualities and intensities based on transcriptome and metabolome data. We detected a coordinated reduction in the expression of genes involved in flavonoid biosynthesis in plants exposed to green and red LEDs, irrespective of the light intensity (Fig. 6). Previous studies have shown that MYB transcription factors, production of anthocyanin pigment 1 (PAP1)/MYB75 and PAP2/MYB90, respectively, play a role in the regulation of anthocyanin biosynthesis in *Arabidopsis thaliana*^44^. In this study, we observed that the expression of PAP2 was down-regulated under green and red LED irradiation at P100 (Fig. 6). On the contrary, blue light at P100 significantly up-regulated the expression of putative candidate genes, *CHS*, *CHI*, *CHI-like 1* (*CHI-L1*), *F3H*, *flavanone 3′-hydroxylase* (*F′3H*), *DFR* and *FLS*. There was no difference in the transcript levels of *PAP1* and *PAP2* genes in plants exposed to blue light or FL.

Although metabolite and transcript profiling revealed coordinated changes in the levels of flavonol derivatives and expression of flavonoid biosynthesis genes (Fig. 6), no significant changes in the transcript levels of chlorogenate biosynthesis genes were observed. These findings suggest that different light qualities and intensities induce complex changes in flavonoid and chlorogenate biosynthesis pathways in lettuce plants. For example, levels of quercetin malonylglucoside and luteolin 7-glucuronide showed a substantial increase following blue light and FL treatments at P300 (Fig. 6), but were unaffected following green and red light treatments. Conversely, as the wavelength of light increased, the amount of dicaffeoyl quinate, one of the major chlorogenates, proportionately decreased, irrespective of the duration of treatment.

## Discussion

In this study, we aimed to investigate metabolic and transcriptional changes and key pathways and processes elicited in lettuce seedlings in response to narrow-band LEDs of different colours and intensities to obtain novel insights into the growth of lettuce and the production of useful metabolites. We performed Illumina-based RNA-Seq analysis to investigate lettuce transcriptome, and a wide range of primary and secondary metabolites were measured using multi-platform MS-based metabolomics^40^. A systems biology approach combining these omics data facilitated the identification of key pathways related to complex and fine-tuned plant responses to different light qualities and intensities. Additionally, we were able to differentiate between the accumulation patterns of various antioxidants, including flavonols, anthocyanins and phenylpropanoids in lettuce seedlings in response to different light conditions (Fig. 6). Jung and colleagues have previously reported a significant correlation between metabolites detected using GC–MS and LC–MS and their antioxidant activity in rice leaves grown under different light conditions using LEDs^45^. Our systems biology approach previously assessed comprehensive metabolic and transcriptional reprogramming of Arabidopsis to UV-B light^46^ and characterised metabolic effects of combined low glutathione with mild oxidative and low phosphorus stress in Arabidopsis^47^. These results suggest that the combination of different light regimes with or without mild stresses intensifies specific metabolic pathways involved in the production of antioxidants.

The quality of seedlings is critical in agriculture, as it determines the growth and yield of transplanted crop plants. High-quality seedlings have desirable morphological characteristics, for example, strong stems, a balanced shoot-to-root ratio and thick, dark green leaves^48^. In addition to these morphological features, specific metabolic products are involved in the promotion of growth after transplanting; for example, seedlings grown under blue light exhibit a high antioxidant activity and grow well after transplanting^16,49^. The leaf size and shape of lettuce seedlings grown under G510 were similar to those of plants grown under B470; however, no accumulation of red pigment was observed under G510 treatment (Fig. 1a). This suggests that plants recognise blue light to produce a red pigment, i.e., anthocyanins (Fig. 6). Multivariate analysis also revealed that although samples exposed to B470 and R680 were clearly separated by PC2, those exposed to FL and green (both G510 and G520) were closer to each other (Fig. 2a). This is consistent with data on rice seedlings irradiated with LEDs^45^. Furthermore, our molecular phenotyping data showed that as small as a 10-nm difference in peak wavelengths of green light (G510 vs. G520) can elicit distinct cellular processes in lettuce plants (Figs 2 and 4). Supplemental red lighting at the beginning of the dark period or blue lighting at end of the dark period at low intensity for 30 min has been shown to increase the yield of spinach, whereas the other two combinations of light quality do not promote growth^10,50^. These observations suggest that seedlings are highly sensitive to the light quality; exposure to different light qualities triggers the production of specific products that may affect plant growth.

Supplementing blue and/or red light with green light has been shown to promote growth in lettuce^22^, lady slipper orchid^51^ and sunflower^24^. Kim and co-workers have shown that lettuce plants grown using red or blue LEDs in combination with green LEDs develop larger leaves with lower thickness than plants exposed to red or blue monochromatic light only^22,23^. Moreover, monochromatic green light has been shown to induce positive phototropism of hypocotyls in Arabidopsis and lettuce^52,53^. These results suggest that green light should be considered as an important wavelength in the light spectrum affecting crop yield. Further investigation is needed to determine whether unique metabolite components in response to different light qualities and intensities are advantageous or disadvantageous for plant growth after transplantation.

Pathway level analysis showed a coordinated down-regulation of flavonoids and the expression of genes in involved in flavonoid biosynthesis in lettuce plants exposed to green LEDs (Fig. 6). Our findings are in agreement with previous reports on lettuce^54^. Furthermore, we showed that green and red light affected the metabolic composition of leaf lettuce, especially the level of flavonoids and phenylpropanoids, which suggests that distinct regulatory mechanisms underlying flavonoid biosynthetic pathways under different light conditions. This implies that UV-B and UV-A/blue light signalling pathways controlling CHS in leaf lettuce are different from phytochrome signalling pathways regulating phenylpropanoids^55,56^. Although plant defence and growth are generally negatively correlated, it is possible that the energy transmitted by green light is effective for balanced production of biomass and secondary metabolites involved in defence mechanisms in leaf lettuce seedlings. Our findings also imply that narrow-band LED lighting can be optimised to better control pigment accumulation and/or gene expressions in lettuce seedlings.

We observed the down-regulation of the *PAP2* gene in parallel with transcriptional coordination of the flavonoid pathway. Additionally, there was no change in *PAP1* expression under green light. These suggest that in lettuce plants exposed to green light, the repression of genes involved in the flavonoid pathway is regulated by PAP2 rather than by PAP1. PAP1 and PAP2 are 93% similar in their amino acid sequence and have similar functions^44^. Our findings are particularly interesting because levels of the major anthocyanin in *PAP2* RNAi lines are similar to those in wild-type Arabidopsis^57^.

A high-quality reference genome for lettuce is now available^58^, and the subsequent functional annotations (e.g. GO terms) facilitated our systems biology study. This improved and curated lettuce gene-set will be highly useful for the development of new crops and functional analysis of the *Compositae* family. In addition to the genome sequence and functional annotations, more large-scale omics data are needed to provide a better estimate of metabolite composition in multiple tissues^59,60^. Since artificial light sources can be used to irradiate plants with various light qualities and intensities at the desired time and for the desired duration, these light sources can efficiently control plant growth and development in plant factories. The use of LEDs in closed-type plant factories and production systems is increasing^16,17,61,62^, and our approach is the first step towards using light as a growth and metabolic regulator for controlled growth and development of crop plants.

## Materials and Methods

### Plant Materials and Growth Conditions

Under white fluorescent light (FL, FLR110H-W1A; Mitsubishi/Osram Co.; Yokohama, Japan), seeds of red leaf lettuce (*Lactuca sativa* L. cv. Banchu red fire; Takii seed, Kyoto, Japan) were pre-germinated (14 h, 14 days, 23 ± 2 °C, 100 μmol·m^−2^·s^−1^ PPFD). Seedlings were supplied with a nutrient solution (Otsuka hydroponic composition, Otsuka Chemical Co. Ltd., Osaka, Japan) adjusted to an electrical conductivity (EC) of 1.2 dS/m and pH 5.8. This nutrient solution contained N, K, Ca, P and Mg at concentrations of 7.6, 3.7, 2.3, 2.0 and 0.9 mmol l^−1^, respectively, and other minor elements. Seedlings were transplanted in cultivation panels in a growth chamber (VB1514; Vötsch, Germany) maintained at 25 °C temperature, 60% relative humidity (RH) and 900 μmol mol^−1^ CO_2_ and supplied with the nutrient solution for the entire duration of experiments. Lettuce plants were irradiated with LEDs supplying different light spectra, including B470, G510, G520 and R680 with peak wavelengths of 470, 510, 524 and 680 nm, respectively (Fig. S12); the LED sources used to supply these wavelengths were ISL-305 × 302-BBBB, ISL-305 × 302-GGGG505, ISL-305 × 302-GGGG525 and ISL-305 × 302-RRRR68, respectively, all of which were purchased from CCS Co., Kyoto, Japan. Lettuce seedlings were continuously irradiated with each light source fitted approximately 30 cm above the cultivation panel for 24 h at PPFD 100 or 300 μmol m^−2^ s^−1^ (P100 and P300, respectively). PPFD was measured with LI-250 light meter (LI-COR, Nebraska, USA) at top of the cultivation panel without plants. The wavelength of the light source was determined with a USB2000 spectrometer (Ocean Optics, Dunedin, FL, USA) (Fig. 1). At 14, 15 and 21 DAS, dry weight (DW) was measured.

### RNA isolation, cDNA library construction and RNA-Seq

Total RNA was isolated from the third leaf of lettuce plants using RNeasy Plant Mini Kit (Qiagen) according to the manufacturer’s instructions. The concentration, integrity and the amount of ribosomal RNA were measured using ND-1000 Spectrophotometer (Thermo Fisher Scientific) and Bioanalyzer 2100 (Agilent Technologies). Oligo(dT) beads were used to isolate poly(A) mRNA from total RNA. Fragmentation buffer was added to fragment mRNA into short pieces. Using these mRNA fragments as templates along with random hexamer primers, we synthesised first-strand cDNA. Second-strand cDNA was synthesised using buffer, dNTPs, RNaseH and DNA polymerase I. The short fragments were then purified with QIAQuick PCR Purification Kit and eluted using EB buffer for repairing ends and adding poly(A) tails. These mRNAs were then ligated with sequencing adapters and size-selected using agarose gel electrophoresis. Suitable fragments were used as templates for PCR amplification and constructing RNA-Seq libraries, which were sequenced using Illumina HiSeq™ 2000. Our RNA-Seq data sets were produced without biological replicates. Same mRNA samples were used for validating RNA-Seq results using qRT-PCR (see below). All data sets generated here are available from the NCBI Sequence Read Archive (SRA; accession number DRA001842).

### RNA-Seq data analysis

Illumina reads that passed quality checks were processed using RobiNA^63^. The remaining short reads were aligned to lettuce genome sequences^58^ with Bowtie^64^. Parameters for mapping reads were -a -m1 -n2 -l28 -e100 -p4. DEGs were identified using DESeq^65^, the data for which was presented as a Venn diagram created using Genevenn (http://genevenn.sourceforge.net/vennresults.php).

### Pathway and functional enrichment analysis of DEGs

The web application Mercator^66^ was used to assign functional MapMan ‘BIN’ ontologies to lettuce protein sequences with a BLAST cutoff ≥ 50 (http://www.plabipd.de/portal/web/guest/mercator-sequence-annotation). Functional BINs were assigned to DEGs using MapMan v 3.5.1R2^67^. To assess whether a functional term was significantly over-represented in the selected genes compared with the set of all genes with MapMan categories, Fisher exact test was used. Probability values for multiple testing problems were adjusted with the Benjamini Hochberg method^68^. GO term enrichment analysis for DEGs was performed with BiNGO^69^. The enriched GO categories were visualised using the Enrichment Map (v2.2.0)^41,42^ and AutoAnnotate^70^.

### Quantitative RT-PCR Validation

Total RNA was extracted using RNeasy Plant Mini Kit (Qiagen). Reverse transcription of each total DNase-treated (Qiagen) RNA sample was performed using the SuperScript III First-Strand Synthesis System for RT-PCR (Invitrogen). qRT-PCR using first-strand cDNA was performed on the ABI StepOnePlus Real Time PCR system (Applied Biosystems) using Fast SYBR Green Master Mix (Applied Biosystems) (Supplementary Table S3). Three biological replicates were used for gene expression validation with qRT-PCR.

### Metabolite profiling

Metabolite profiling was conducted using GC–MS and LC–MS as described previously^36–39^ with slight modifications. Metabolite levels of lettuce plants exposed to blue, green and red LEDs were measured using frozen leaf samples (see Supplementary Document S1 for details). Principal component analysis (PCA) and O2PLS-DA were performed using SIMCA-P version 14 (Umetrics, Umea, Sweden) with log_10_ transformation and autoscaling. The O2PLS-DA model was validated by cross validation-analysis of variance (CV-ANOVA); the *p*-value cut-off was *p* < 0.01. Differentially accumulated metabolites were identified using the LIMMA package^71^, which generates FDR-adjusted *p*-values for multiple testing problems^68^. Results were considered statistically significant at FDR < 0.05. Significantly changed metabolites were presented in a Venn diagram created using VennDiagram package^72^. All metabolite profiling experiments included 5–6 biological replicates.

### Data availability

All raw short reads in FASTQ format are available for download at the DDBJ Sequence Read Archive under accession number DRA001842.

## Electronic supplementary material

Supplementary Information

Supplementary Table S1

Supplementary Table S2

Supplementary Table S3
