# Supplementary material for: Metabolic Reprogramming in Leaf Lettuce Grown Under Different Light Quality and Intensity Conditions Using Narrow-Band LEDs
Source: Sci Rep. 2018 May 21;8:7914. doi: 10.1038/s41598-018-25686-0 (PMC5962576; doi:10.1038/s41598-018-25686-0)
Supplement: Supplementary file 1 — Supplementary Information [file 41598_2018_25686_MOESM1_ESM.pdf]

Metabolic Reprogramming in Leaf Lettuce Grown Under Different Light Quality and Intensity Conditions Using Narrow-Band LEDs

Authors:

Kazuyoshi Kitazaki[1†], Atsushi Fukushima[2], Ryo Nakabayashi[2], Yozo Okazaki[2], Makoto Kobayashi[2], Tetsuya Mori[2], Tomoko Nishizawa[2], Sebastian Reyes-Chin-Wo[3], Richard W. Michelmore[3], Kazuki Saito[2,4], Kazuhiro Shoji[1*], Miyako Kusano[2,5*]

Affiliation:

[1]*Central Research Institute of Electric Power Industry, Abiko, Chiba, 270-1194, Japan*

[2]*RIKEN Center for Sustainable Resource Science, Yokohama, Kanagawa, 230-0045, Japan*

[3]*UC Davis Genome Center, Davis, California, 95616, USA*

[4]*Graduate School of Pharmaceutical Sciences, Chiba University, Chiba, Chiba, 263-8522, Japan*

[5]*Graduate School of Life and Environmental Sciences, University of Tsukuba, Tsukuba, Ibaraki, 305-8572, Japan*

[†]*Present address: Research Faculty of Agriculture, Hokkaido University, Sapporo, Hokkaido, 060-8589, Japan*

Kazuyoshi Kitazaki and Atsushi Fukushima contributed equally to this work.

*Correspondence: shoji@criepi.denken.or.jp and kusano.miyako.fp@u.tsukuba.ac.jp

Figure S1. Metabolomic characterisation of leaf lettuce exposed to blue and red LEDs. (**a**) Principal component analysis (PCA) score scatter plot of metabolite profiles of lettuce samples in response to blue (B470, blue squares), red (R680, red inverted

triangles) and fluorescent light (FL, empty triangles) treatment. Metabolite profiles were obtained by combining GC-MS (GC), LC-MS (LC) and LC-ion trap-MS (IT) data. (**b**) Orthogonal Projection to Latent Structures Discriminant Analysis (O2PLS-DA) score scatter plot (upper part) and loading plot (bottom part) of metabolite profiles of lettuce samples by blue, red and FL treatment. Lettuce seedlings were grown under continuous blue, red or FL at 100 μmol·m^{-2}·s^{-1} photosynthetic photon flux density (PPFD) for 1 week. Metabolites detected by GC, IT and LC are indicated using yellow circles, green rectangles and light blue triangles, respectively, in the loading plot. Data matrix consists of 19 samples and 235 identified/annotated peaks with three response factors as represented by $M8.DA(1)–(3). Cross validation-analysis of variance (CV-ANOVA) was performed to assess model reliability. CV-ANOVA *p*-value = 3.52E-7. PC, principal component; Pred PC, predictive principal component.

Figure S2. Box plots showing changes in the content of primary and secondary metabolites in the third leaf of lettuce plants grown under variable light quality for different durations at PPFD of 300 μmol·m^{-2}·s^{-1} (P300).

Black horizontal lines in the boxes represent the median, whereas the top and bottom of the boxes represent the upper (75th) and lower quartiles (25th), respectively. Circles show potential outliers. Six biological replicates were performed per condition. Values are normalised relative to those obtained from fluorescent light (FL) treatment (control). **a**, sugars and sugar alcohol; **b**, TCA-cycle intermediates; **c**, Gln, Glu and Asp; **d**, stress-related metabolites; **e**, branched-chain and aromatic amino acids; **f**, Ser/Gly and sulphur metabolism; **g**, chlorogenate and flavonoid metabolism; **h**, carotenoids and chlorophylls; **i**, sesquiterpene lactones. G6P, glucose-6-phosphate; F6P, fructose-6-phosphate; 2OG, 2-oxogluatarate; Gln, glutamine; Glu, glutamic acid; Asp, aspartic

acid; GABA, gamma-aminobutyric acid; Ser, serine; Gly, glycine; and OAS, *O*-acetylserine.

Figure S3. Principal component analysis score scatter plots of leaf lettuce seedlings grown under different light qualities and intensities. Metabolic responses of leaves treated with (**a**) short-term (1 day) and (**b**) long-term (7 days) exposure to different light qualities [FL, fluorescent light; B470, blue (470 nm); G510 and G520, green (510 and 524 nm, respectively); and R680, red (680 nm)] and intensities (P100 and P300). Each symbol indicates an independent plant sample in the score scatter plot (biological replicates, $n = 6$).

Figure S4. Venn diagrams showing differentially accumulated metabolites. We analysed differentially accumulated metabolites from the lettuce samples irradiated different wavelength LEDs and intensities. We identified significantly changes in metabolite levels compared to FL condition using LIMMA [71]. (**a**) Increased metabolites and (**b**) decreased metabolites after 7 days of irradiation are shown. Significance level was set as follows, $|\log_2\text{-foldchange}| >= 1$, false discovery rate (FDR) < 0.05. FL, fluorescent lamp.

Figure S5. Sequencing statistics.

Q20, quality score of 20; total clean nucleotides = total reads 1 × read 1 size + total reads 2 × read 2 sizes.

Figure S6. Venn diagram representing differentially expressed genes (DEGs) overlapping among the samples irradiated with different LEDs and with fluorescent light (FL; control).

The cutoff for significant DEGs was FDR < 0.05 by LIMMA [71]. DEGs were visualised with Venny (http://bioinfogp.cnb.csic.es/tools/venny/).

Figure S7. Overview of the transcript profiles associated with blue (B470; 470 nm) and green (G510; 510 nm) light responses.

This information is based on Enrichment Map [41,42] (see the figure legend of Fig. 4 for details).

Figure S8. Overview of the transcript profiles associated with blue (B470; 470 nm) and green (G520; 524 nm) light responses.

This information is based on Enrichment Map [41,42] (see the figure legend of Fig. 4 for details).

Figure S9. Overview of the transcript profiles associated with red (R680; 680 nm) and green (G520; 524 nm) light responses.

This information is based on Enrichment Map [41,42] (see the figure legend of Fig. 4 for details).

Figure S10. Overview of gene expression patterns of lettuce plants irradiated with green LED (GL) at PPFD of 300 μmol·m^{-2}·s^{-1} (P300).

We used MAPMAN software (http://mapman.gabipd.org/web/guest/mapman) [67]. (**a**) G510 (peak wavelength 510 nm). (**b**) G520 (peak wavelength 524 nm). Fold-changes are in gene expression are normalised relative to white fluorescent light (FL) treatment. Statistically significant changed in expression are identified by colour: red = up-regulated by GL treatment and blue = down-regulated by GL treatment.

Figure S11. qRT-PCR validation of RNA-Seq data obtained from lettuce plants grown under high-intensity light (P300). Plants of red leaf lettuce were exposed to different light qualities [FL = fluorescent lamp, B470 = blue (470 nm), G510 and G520 = green (510 and 524 nm, respectively), R680 = red (680 nm) LEDs] for 24 h. Expression levels of six genes involved in flavonoid biosynthesis were investigated

using qRT-PCR, including *phenylalanine ammonia-lyase* (*PAL*), *chalcone synthase* (*CHS*), *flavanone 3-hydroxylase* (*F3H*), *dihydroflavonol 4-reductase* (*DFR*), *anthocyanidin synthase* (*ANS*) and *UDP-glucose:flavonoid 3*-O-*glucosyltransferase* (*UFGT*). Three biological replicates were performed for each gene. Error bars represent standard deviation. Welch's *t*-test was used to calculate *p*-values. Statistically significant differences in expression levels are indicated using an asterisk ($p < 0.05$). Gene-specific primers used for qRT-PCR are listed in Supplementary Table S3. d, day.

Figure S12. Spectral photon flux distributions of lights used for growing leaf lettuce in this study.

(**a**) White fluorescent light (FL). (**b**) LEDs, B470: peak wavelength of 470 nm, G510: peak wavelength of 510 nm, G520: peak wavelength of 524 nm, R680: peak wavelength of 680 nm. The total photosynthetic photon flux (PPF) was 100 $\mu mol \cdot m^{-2} \cdot s^{-1}$ for each treatment.

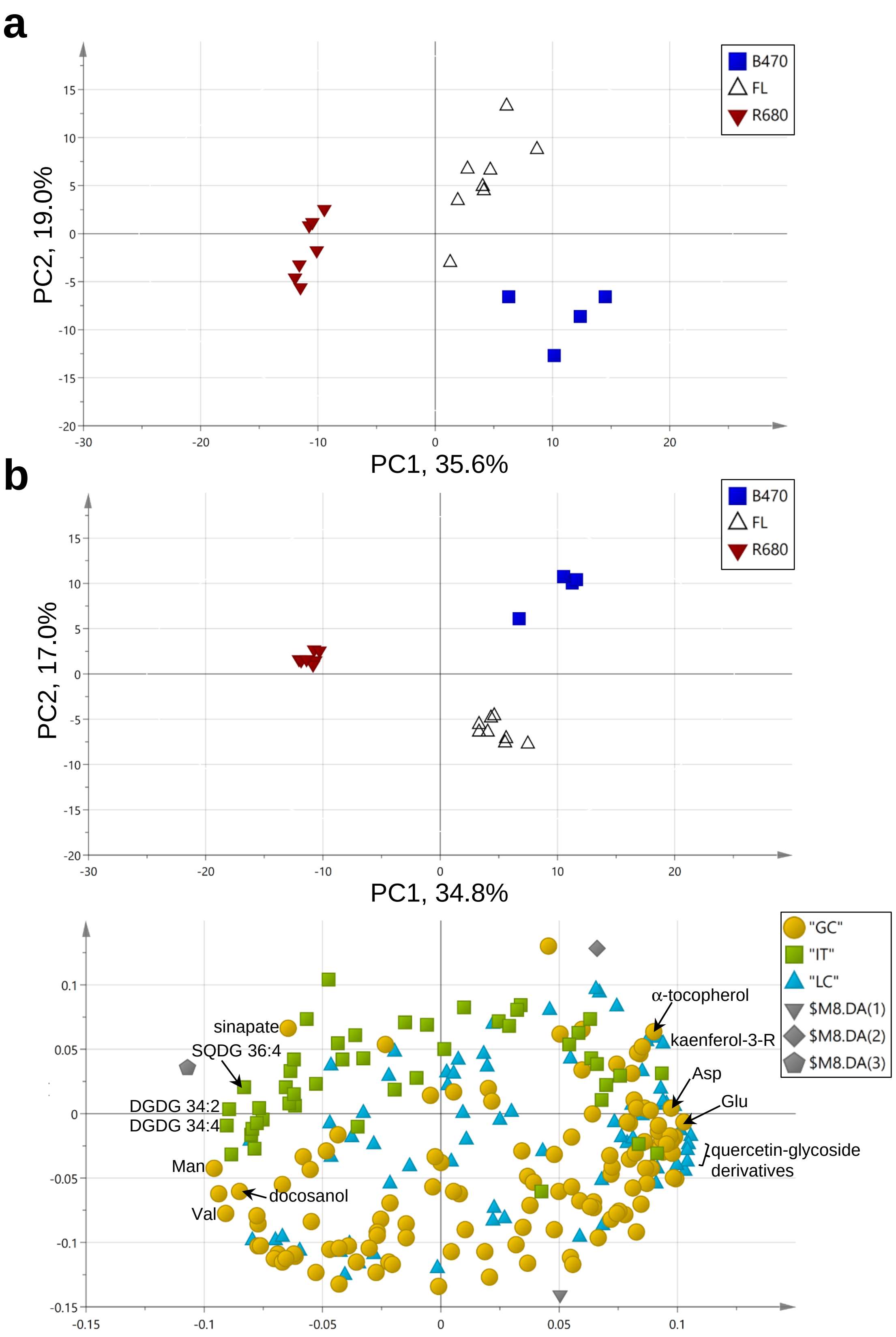

a
B470
FL
R680
PC2, 19.0%
PC1, 35.6%
b
B470
FL
R680
PC2, 17.0%
PC1, 34.8%
"GC"
"IT"
"LC"
$M8.DA(1)
$M8.DA(2)
$M8.DA(3)
α-tocopherol
kaenferol-3-R
Asp
Glu
quercetin-glycoside derivatives
sinapate
SQDG 36:4
DGDG 34:2
DGDG 34:4
Man
docosanol
Val

Figure S1

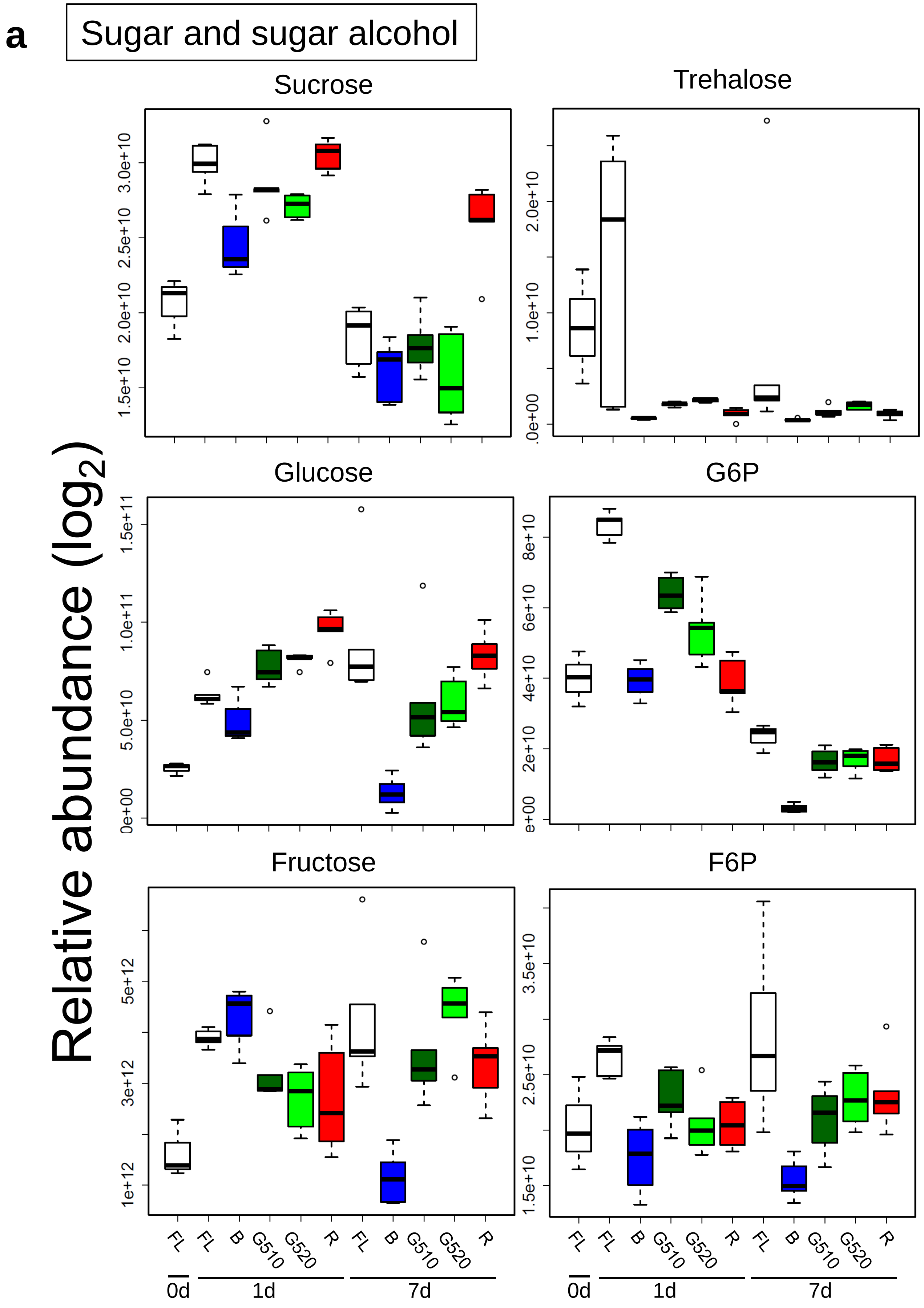
a
Sugar and sugar alcohol
Relative abundance (log2)
Sucrose
Trehalose
Glucose
G6P
Fructose
F6P
FL
FL
B
G510
G520
R
FL
B
G510
G520
R
0d
1d
7d

Figure S2

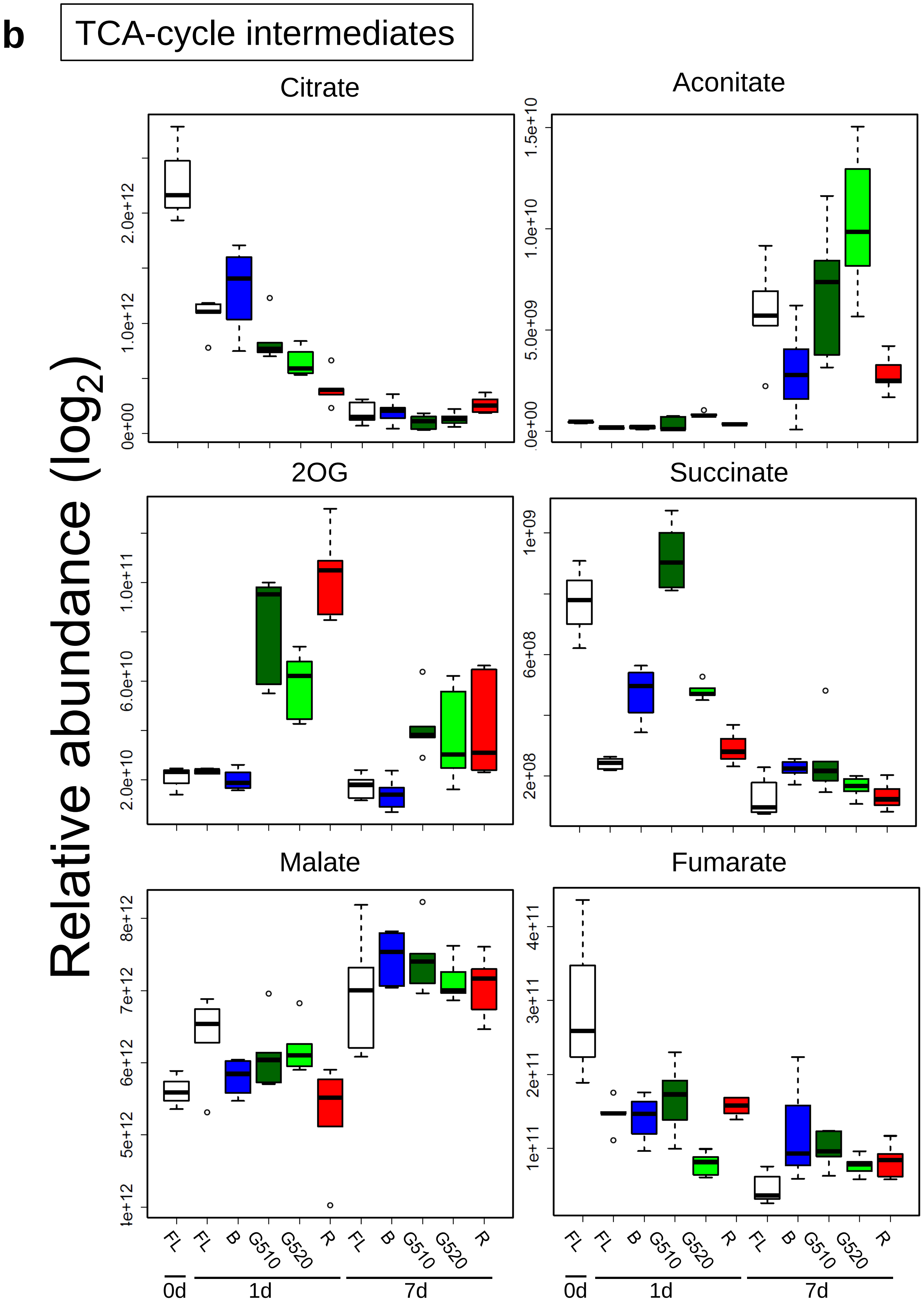

Figure S2 (continued)

c Gln, Glu, and Asp

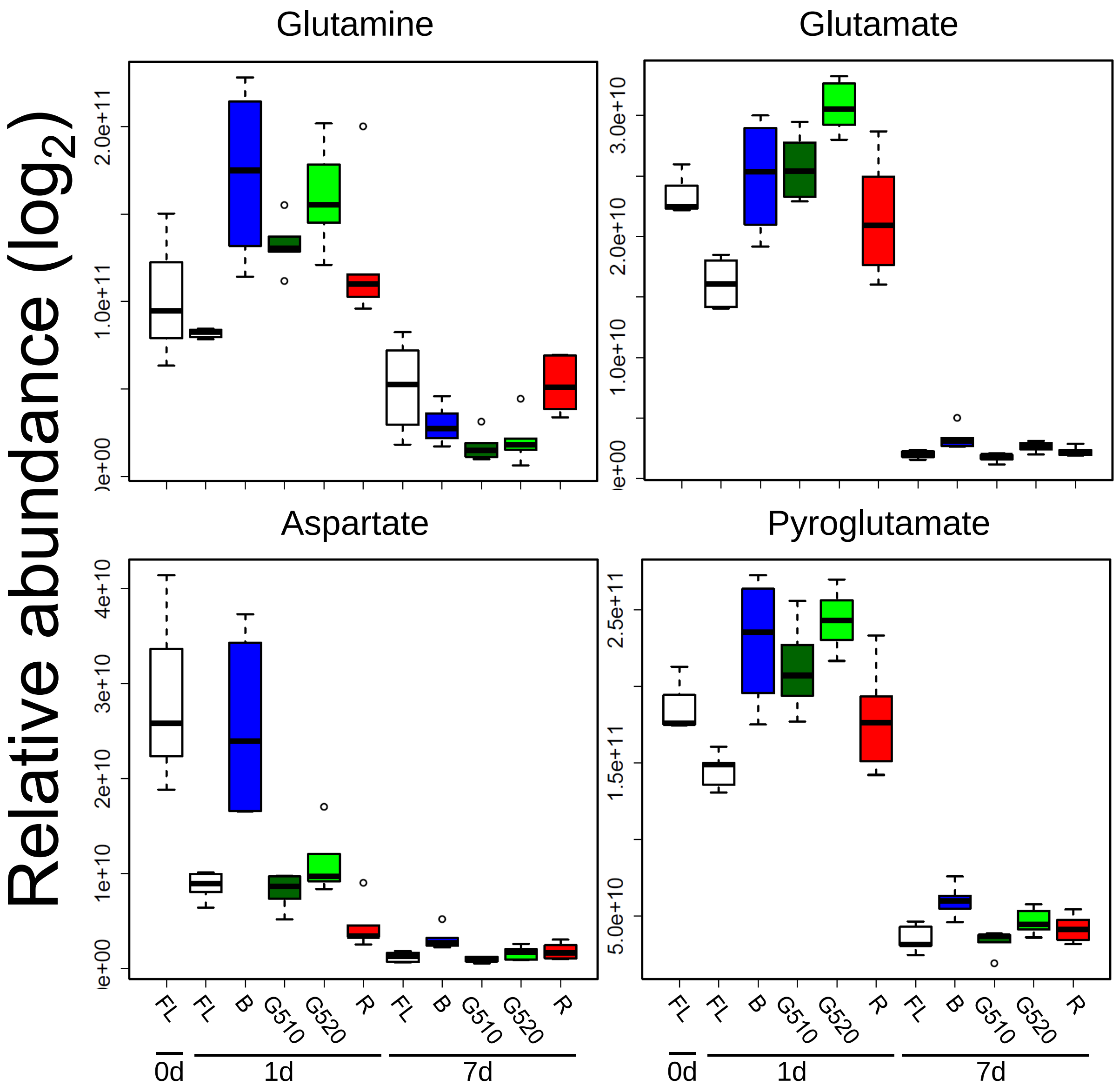

Figure S2 (continued)

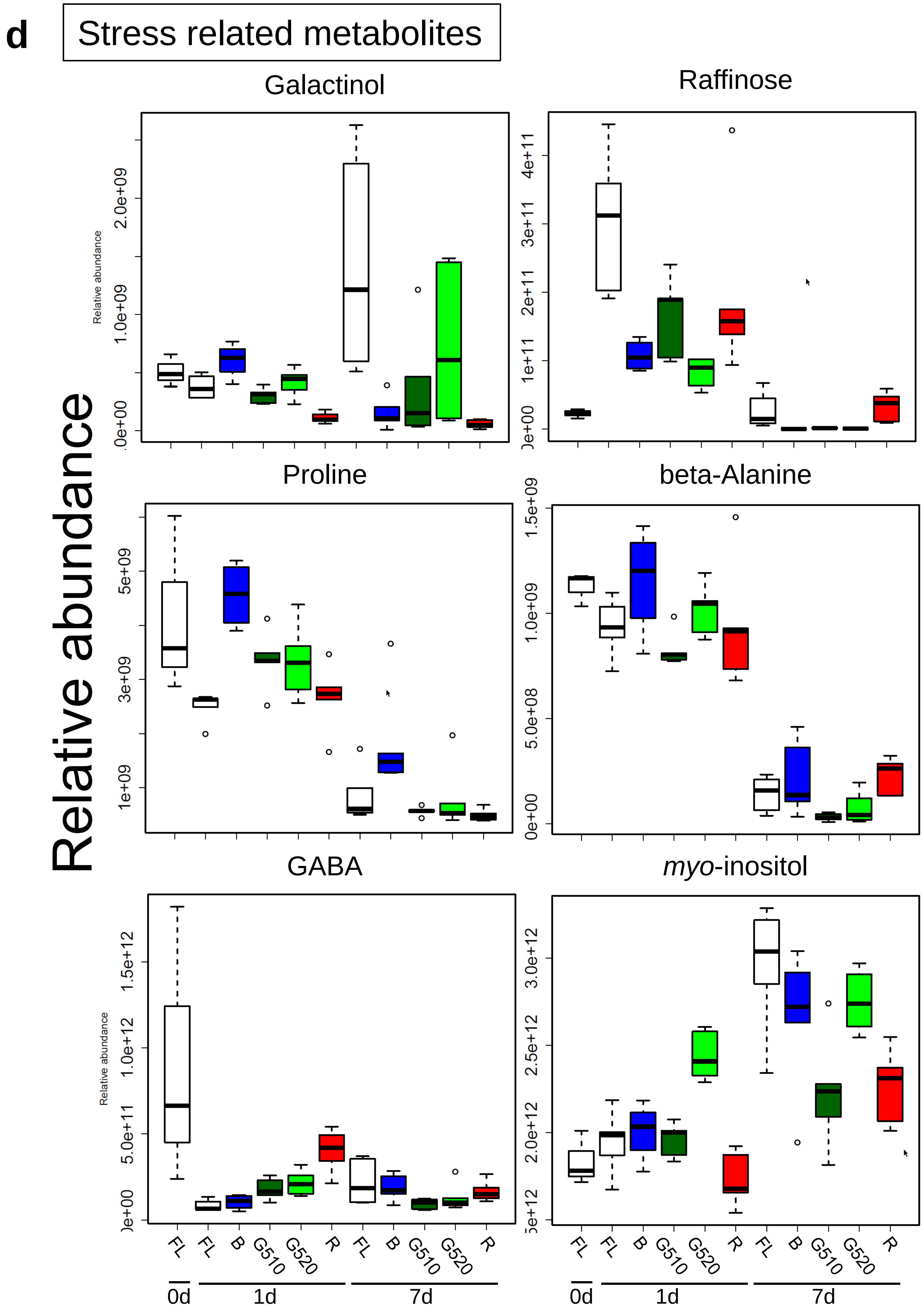

Figure S2 (continued)

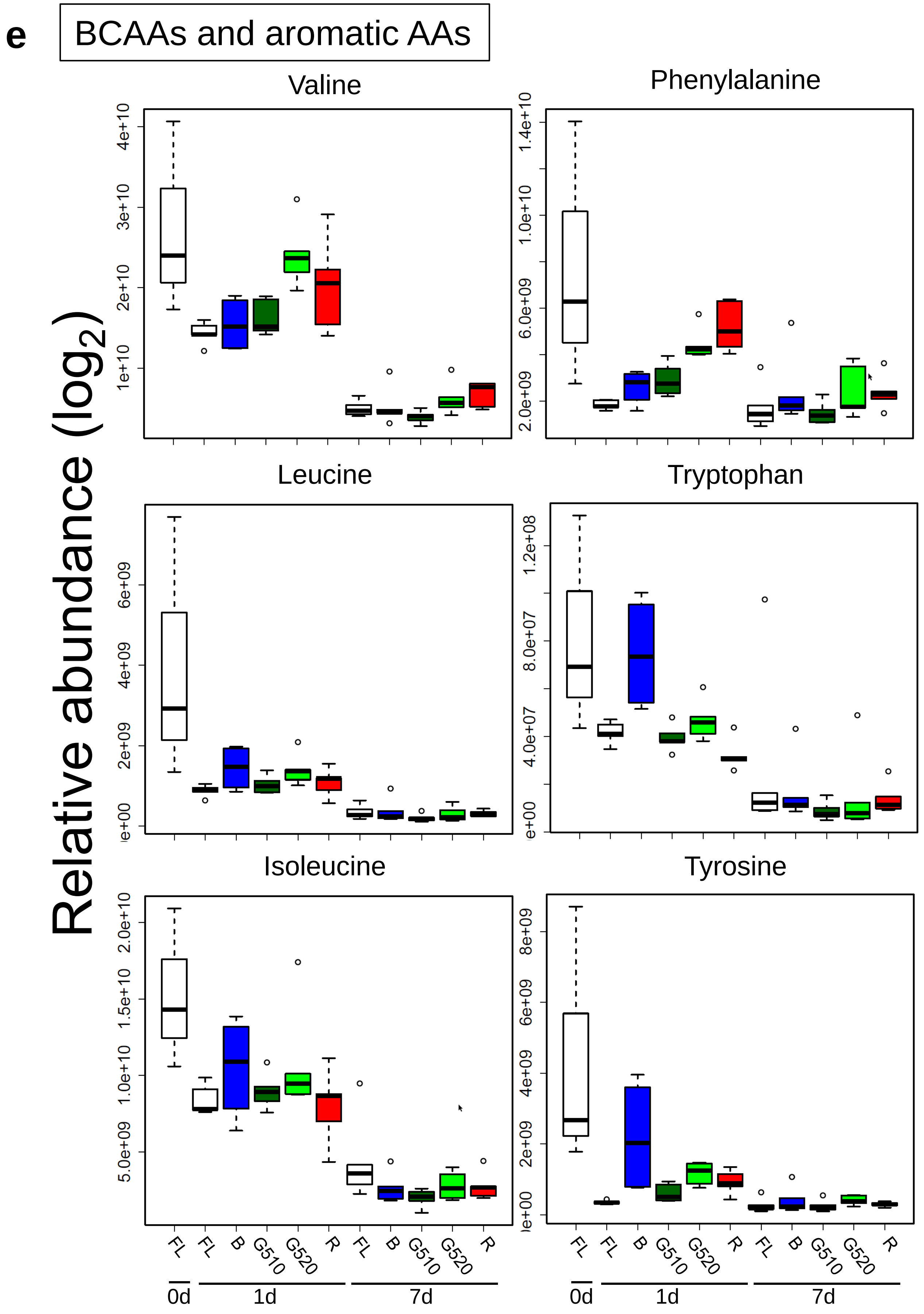

Figure S2 (continued)

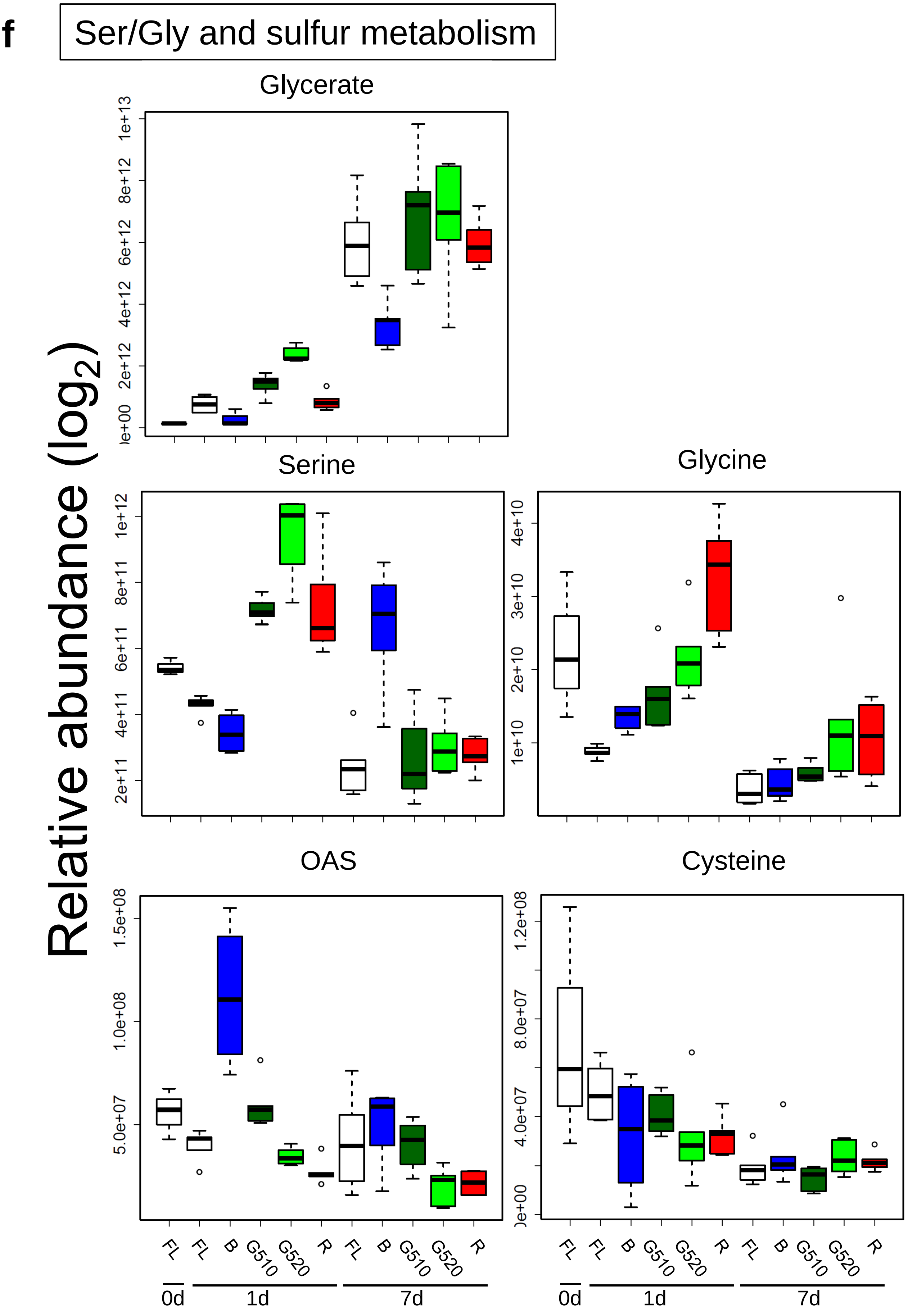

Figure S2 (continued)

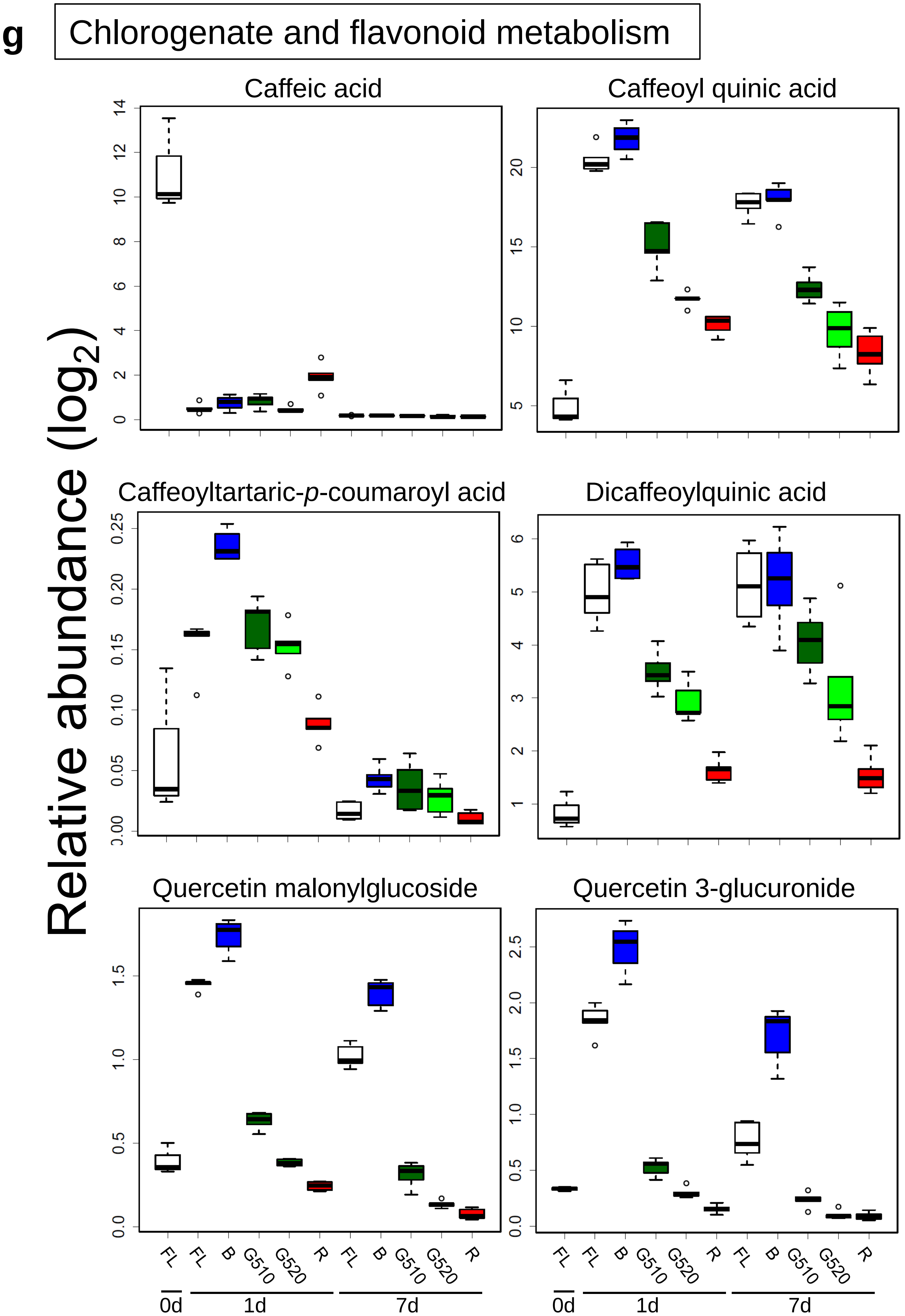

Figure S2 (continued)

h

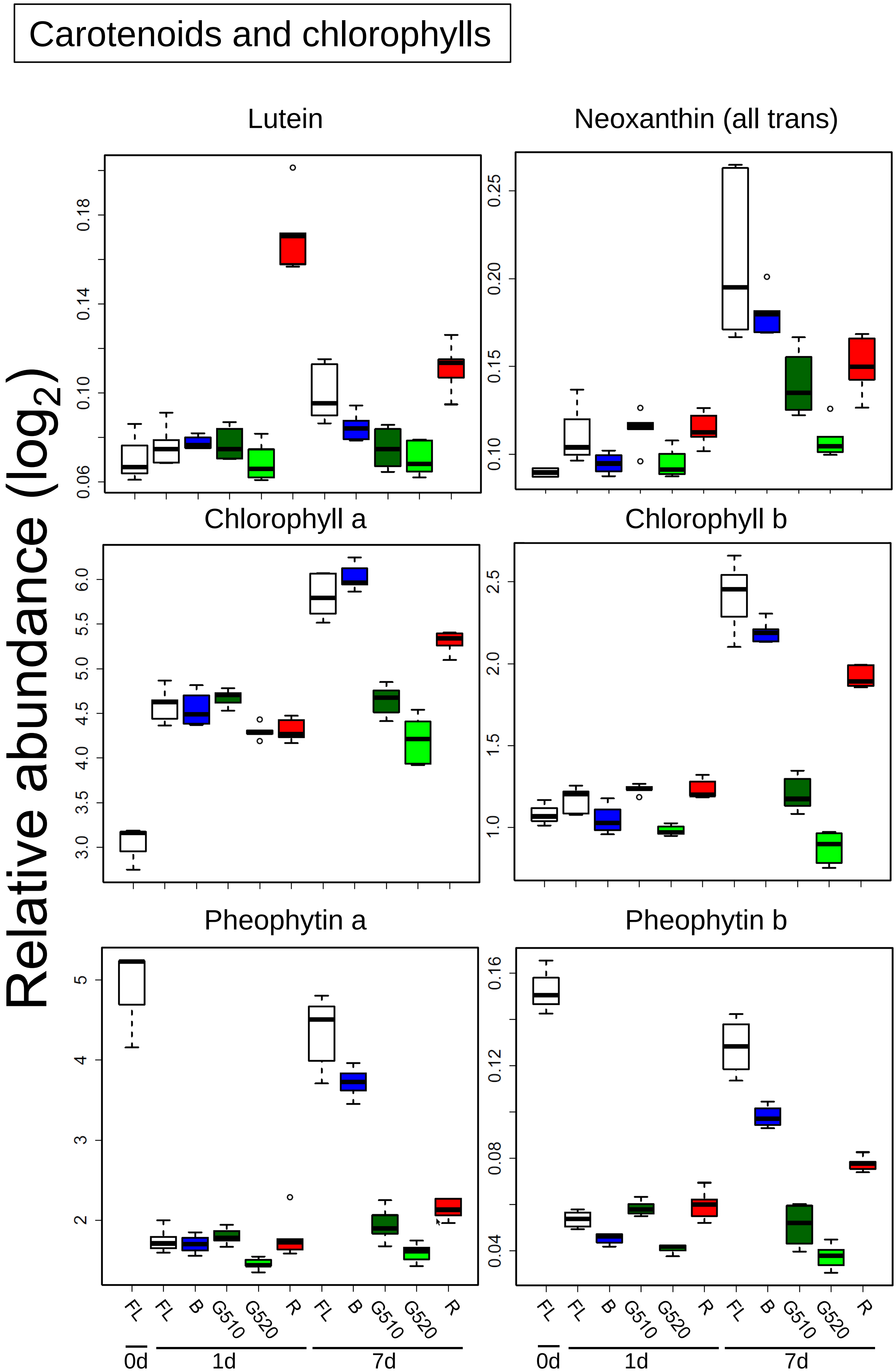

Figure S2 (continued)

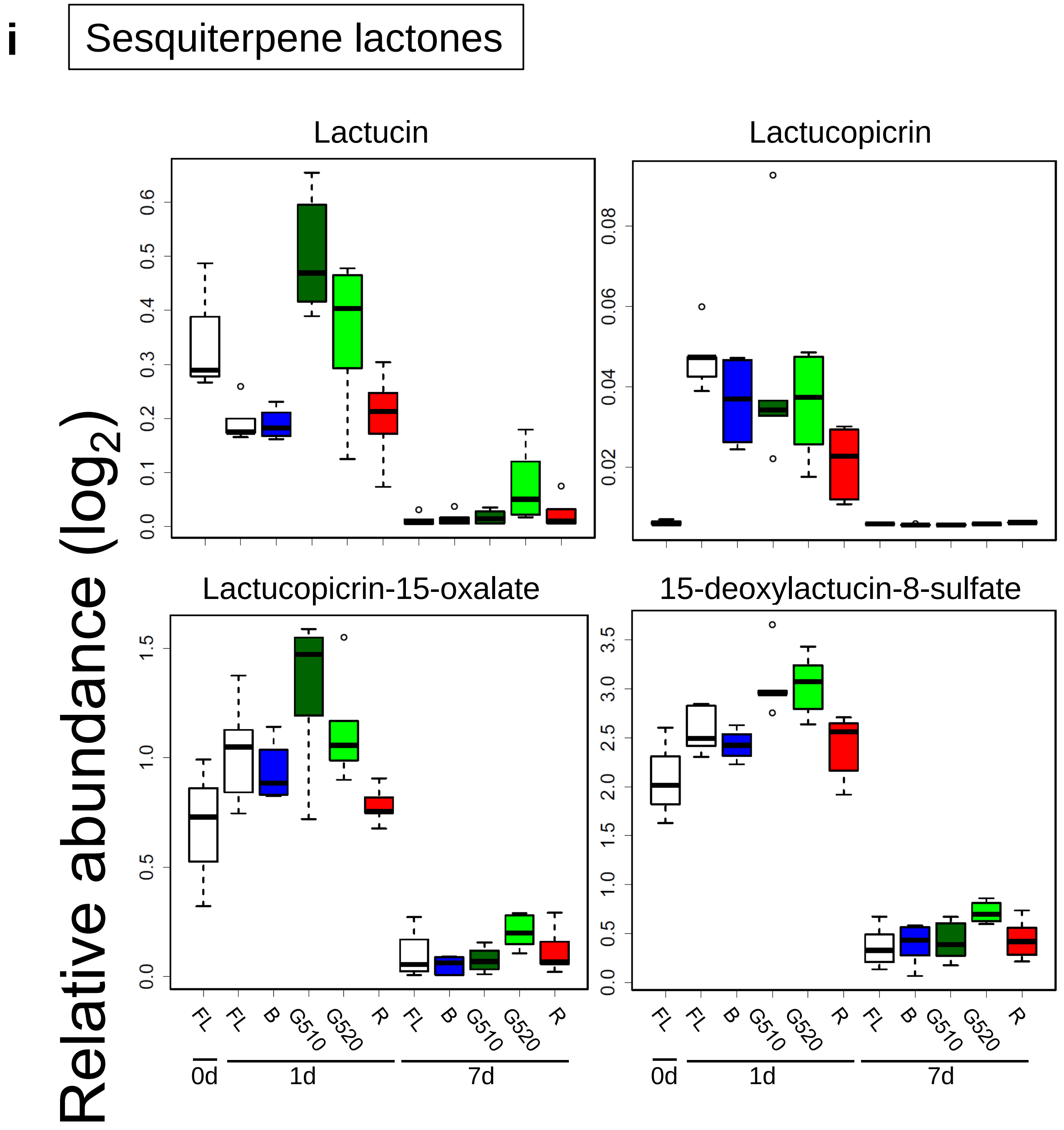

Figure S2 (continued)

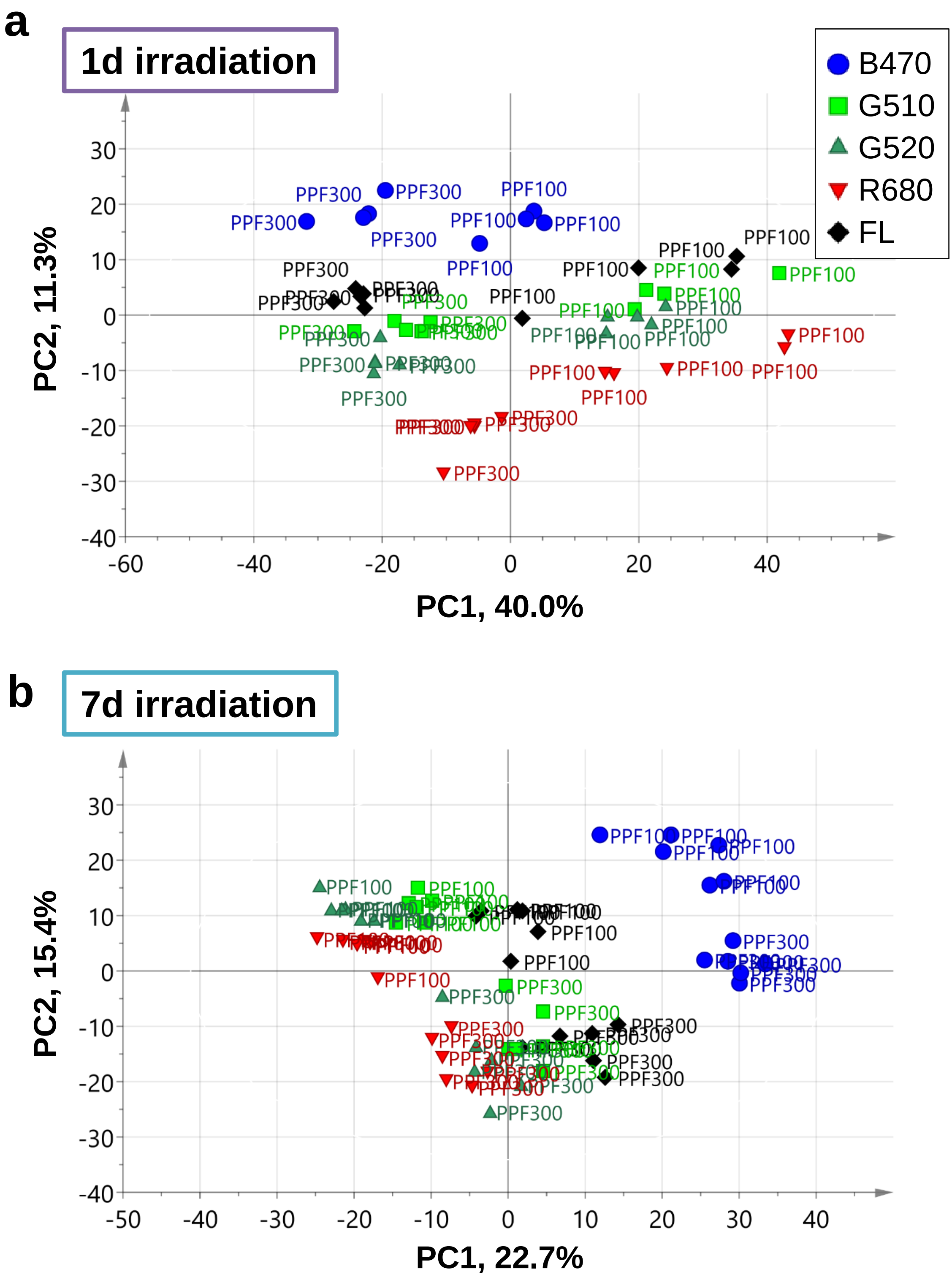
a
1d irradiation
B470
G510
G520
R680
FL
PC2, 11.3%
PC1, 40.0%
b
7d irradiation
PC2, 15.4%
PC1, 22.7%

Figure S3

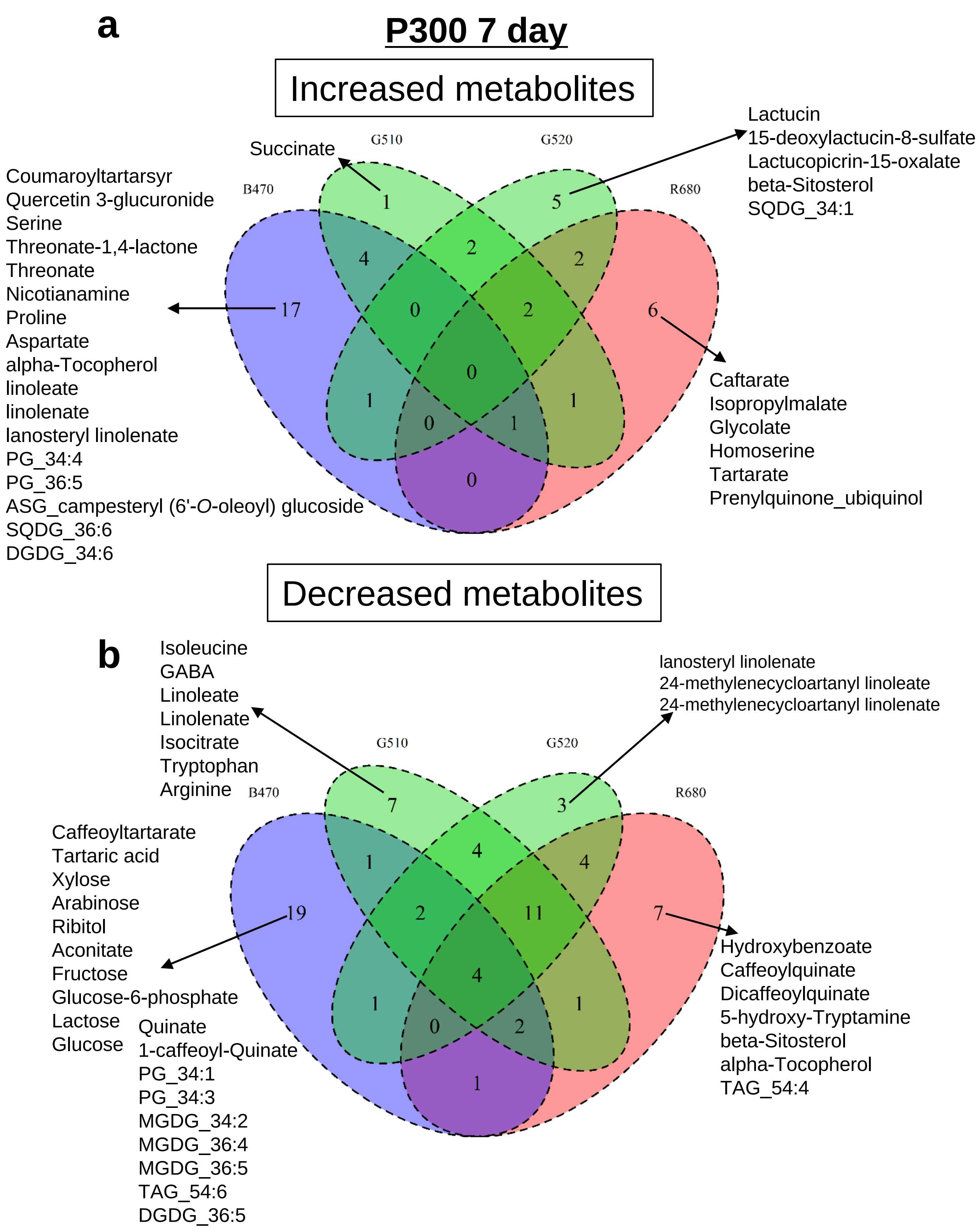
a
P300 7 day
Increased metabolites
B470
G510
G520
R680
Succinate
Coumaroyltartarsyr
Quercetin 3-glucuronide
Serine
Threonate-1,4-lactone
Threonate
Nicotianamine
Proline
Aspartate
alpha-Tocopherol
linoleate
linolenate
lanosteryl linolenate
PG_34:4
PG_36:5
ASG_campesteryl (6'-O-oleoyl) glucoside
SQDG_36:6
DGDG_34:6
Lactucin
15-deoxylactucin-8-sulfate
Lactucopicrin-15-oxalate
beta-Sitosterol
SQDG_34:1
Caftarate
Isopropylmalate
Glycolate
Homoserine
Tartarate
Prenylquinone_ubiquinol
17
1
5
6
4
2
2
0
2
0
1
1
0
1
0
Decreased metabolites
b
B470
G510
G520
R680
Isoleucine
GABA
Linoleate
Linolenate
Isocitrate
Tryptophan
Arginine
lanosteryl linolenate
24-methylenecycloartanyl linoleate
24-methylenecycloartanyl linolenate
Caffeoyltartarate
Tartaric acid
Xylose
Arabinose
Ribitol
Aconitate
Fructose
Glucose-6-phosphate
Lactose
Glucose
Quinate
1-caffeoyl-Quinate
PG_34:1
PG_34:3
MGDG_34:2
MGDG_36:4
MGDG_36:5
TAG_54:6
DGDG_36:5
Hydroxybenzoate
Caffeoylquinate
Dicaffeoylquinate
5-hydroxy-Tryptamine
beta-Sitosterol
alpha-Tocopherol
TAG_54:4
19
7
3
7
1
4
4
2
11
4
1
1
0
2
1

Figure S4

RIKEN sample name	Total Raw Reads	Total Clean Reads	Total Clean Nucleotides (nt)	Q20%	N%	G+C%
P100 FL	31,803,598	27,166,164	2,444,954,760	94.74%	0.00%	46.13%
P300 FL	31,464,142	27,485,016	2,473,651,440	94.74%	0.00%	45.97%
P300 470nm	32,000,000	28,095,976	2,528,637,840	94.68%	0.00%	46.17%
P300 510nm	31,823,538	27,344,692	2,461,022,280	94.79%	0.00%	45.56%
P300 520nm	29,982,232	25,952,786	2,335,750,740	94.65%	0.00%	46.53%
P270 680nm	29,646,750	25,690,178	2,312,116,020	94.80%	0.00%	46.41%

Figure S5

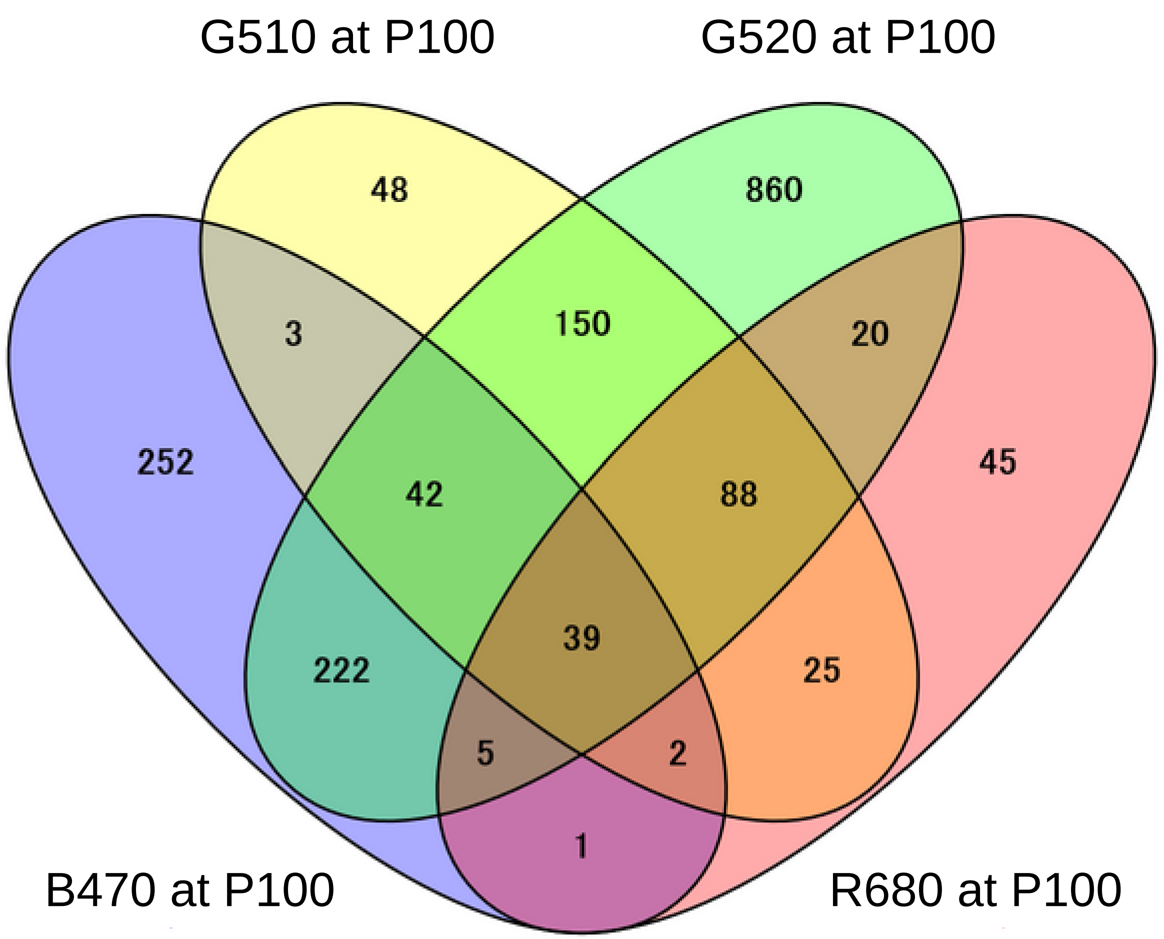
G510 at P100
G520 at P100
48
860
3
150
20
252
42
88
45
39
222
25
5
2
1
B470 at P100
R680 at P100

Figure S6

B470 vs. G510 [FL P100]

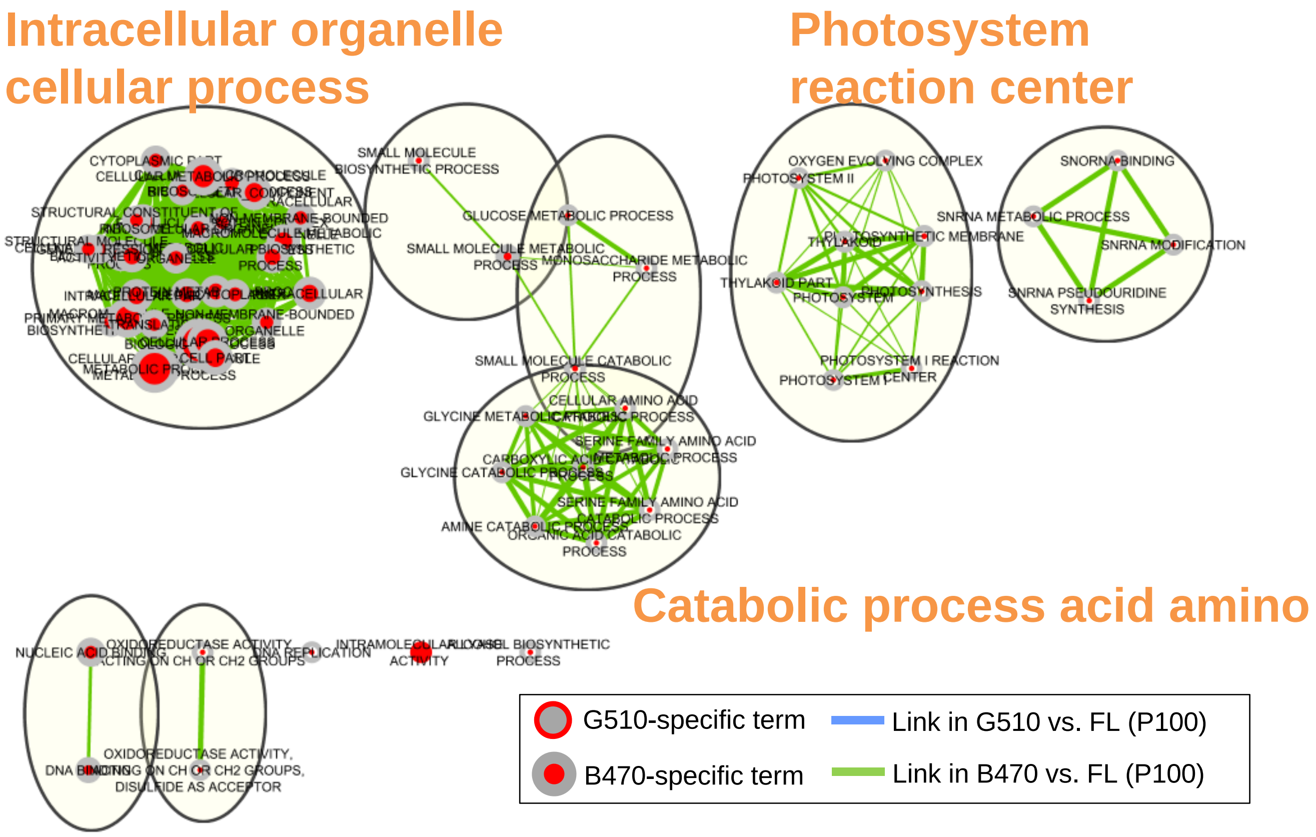

Figure S7

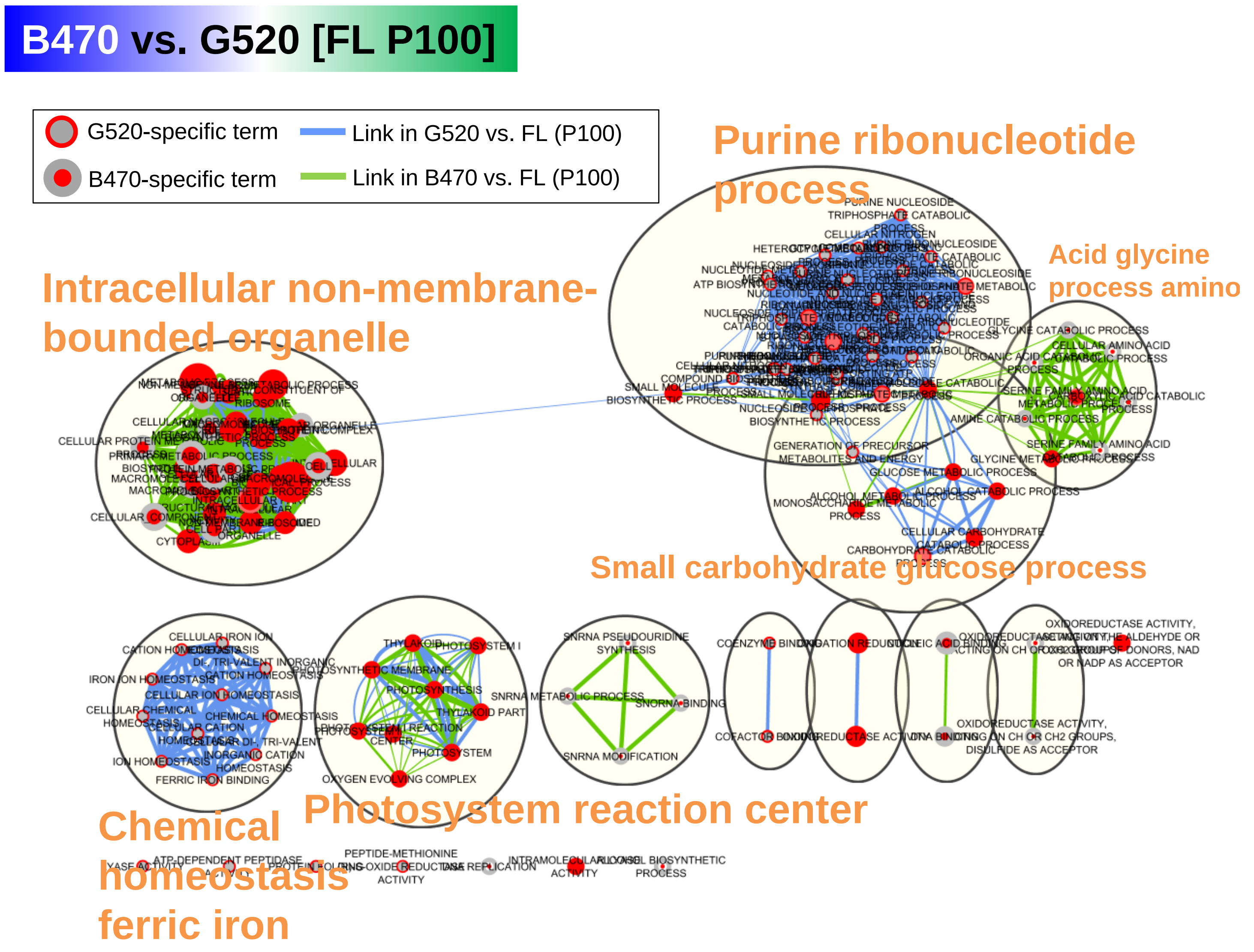

B470 vs. G520 [FL P100]
G520-specific term
B470-specific term
Link in G520 vs. FL (P100)
Link in B470 vs. FL (P100)
Purine ribonucleotide process
Acid glycine process amino
Intracellular non-membrane-bounded organelle
Small carbohydrate glucose process
Chemical homeostasis ferric iron
Photosystem reaction center

Figure S8

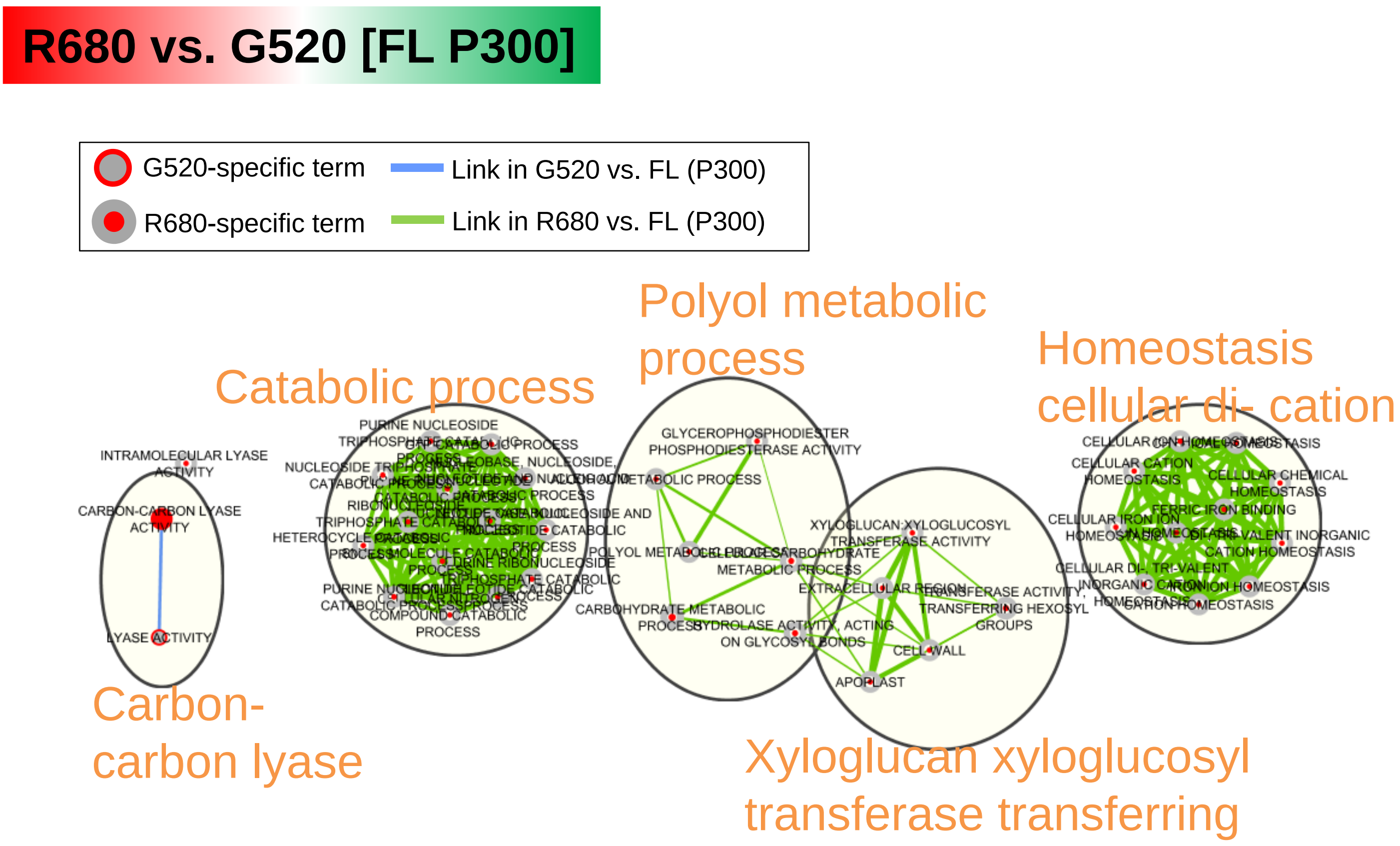
R680 vs. G520 [FL P300]
G520-specific term
R680-specific term
Link in G520 vs. FL (P300)
Link in R680 vs. FL (P300)
Catabolic process
Polyol metabolic process
Homeostasis cellular di- cation
Carbon-carbon lyase
Xyloglucan xyloglucosyl transferase transferring
INTRAMOLECULAR LYASE ACTIVITY
CARBON-CARBON LYASE ACTIVITY
LYASE ACTIVITY
GLYCEROPHOSPHODIESTER PHOSPHODIESTERASE ACTIVITY
CARBOHYDRATE METABOLIC PROCESS
CELL WALL
APOPLAST
FERRIC IRON BINDING

Figure S9

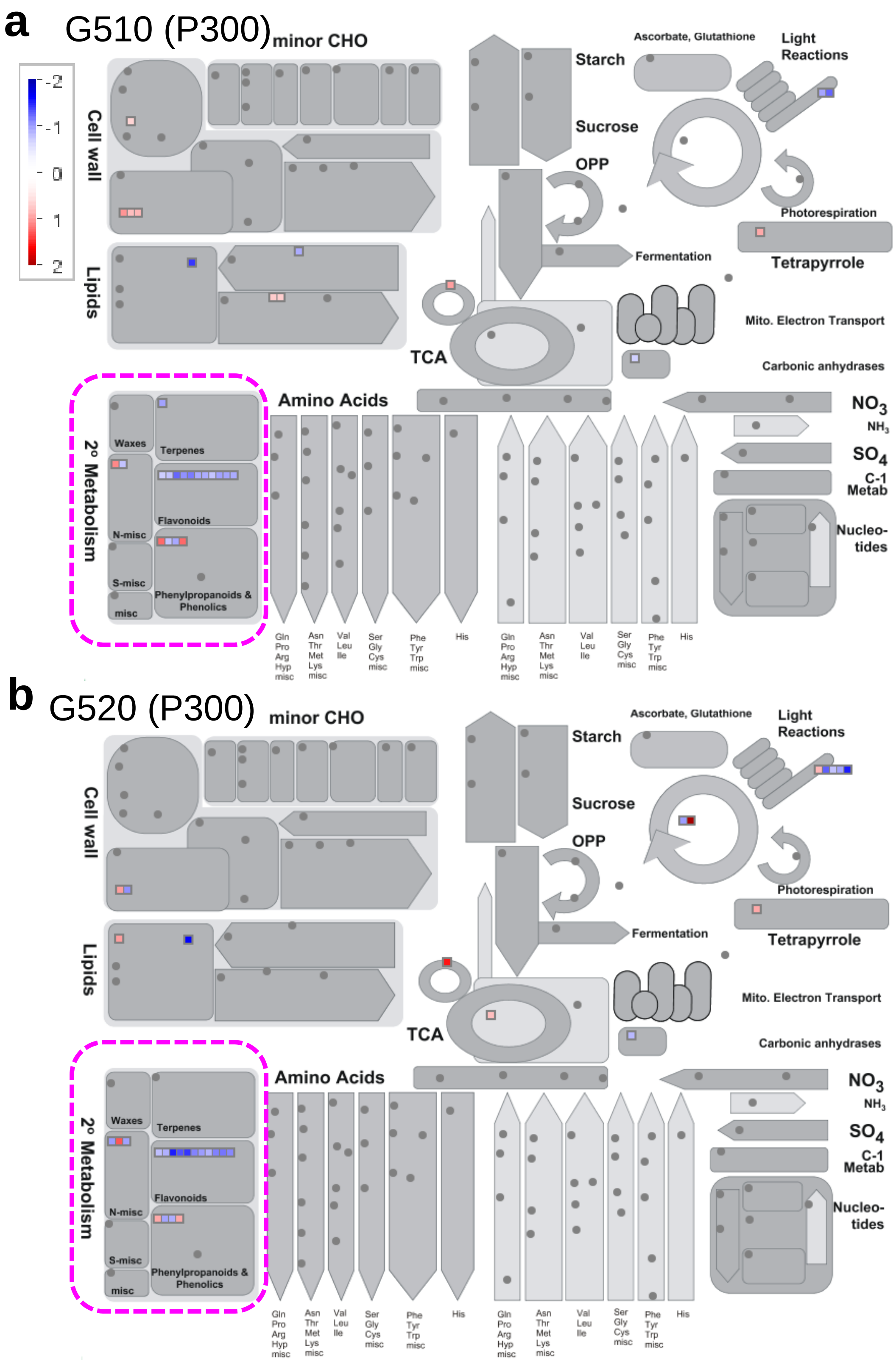
a
G510 (P300)
b
G520 (P300)
minor CHO
Cell wall
Lipids
Starch
Sucrose
OPP
Ascorbate, Glutathione
Light Reactions
Photorespiration
Tetrapyrrole
Fermentation
TCA
Mito. Electron Transport
Carbonic anhydrases
Amino Acids
2° Metabolism
Waxes
Terpenes
Flavonoids
N-misc
S-misc
misc
Phenylpropanoids & Phenolics
NO3
NH3
SO4
C-1 Metab
Nucleo-tides
Gln Pro Arg Hyp misc
Asn Thr Met Lys misc
Val Leu Ile
Ser Gly Cys misc
Phe Tyr Trp misc
His
-2
-1
0
1
2

Figure S10

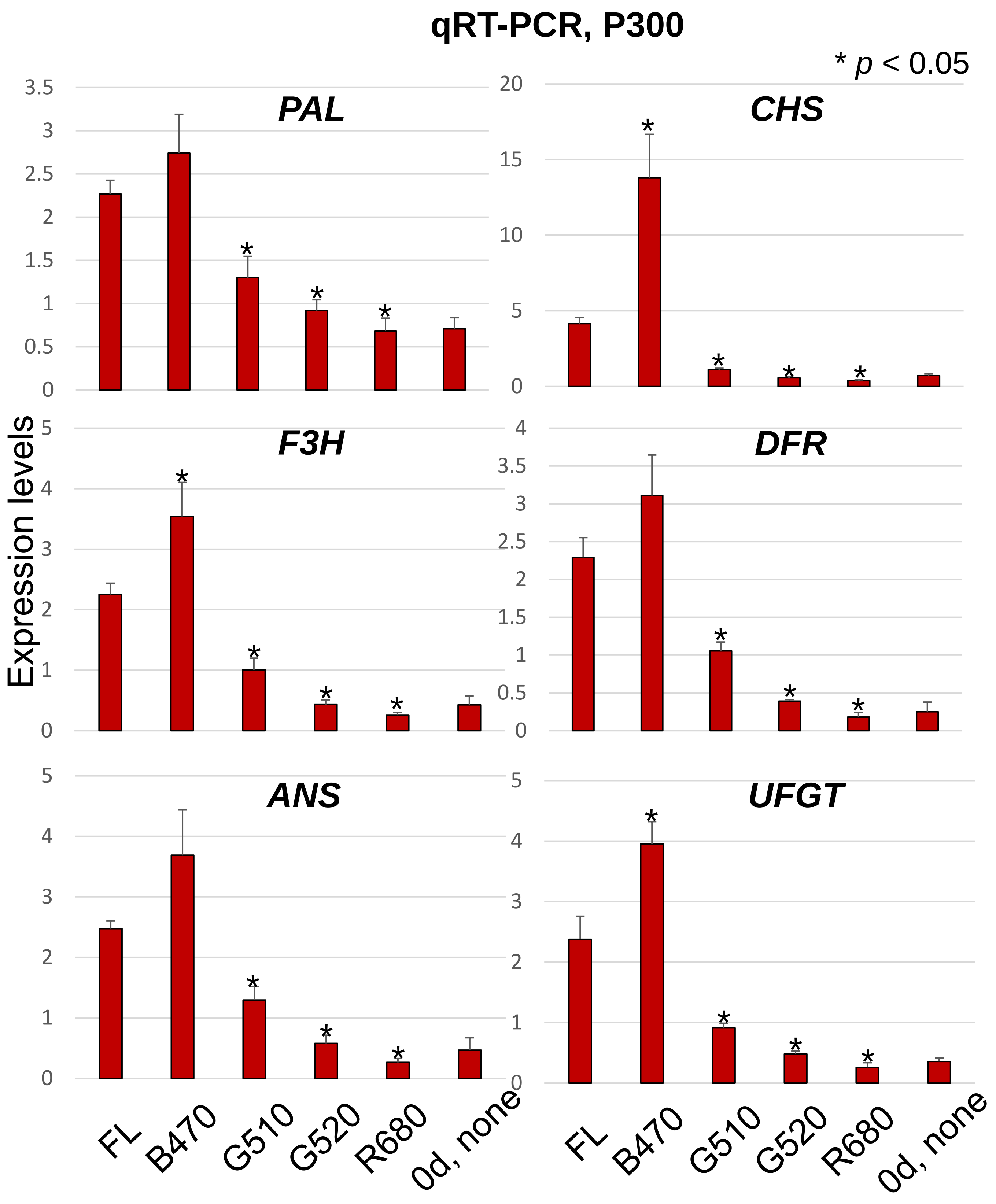

qRT-PCR, P300
* p < 0.05
PAL
CHS
F3H
DFR
ANS
UFGT
Expression levels
FL
B470
G510
G520
R680
0d, none

Figure S11

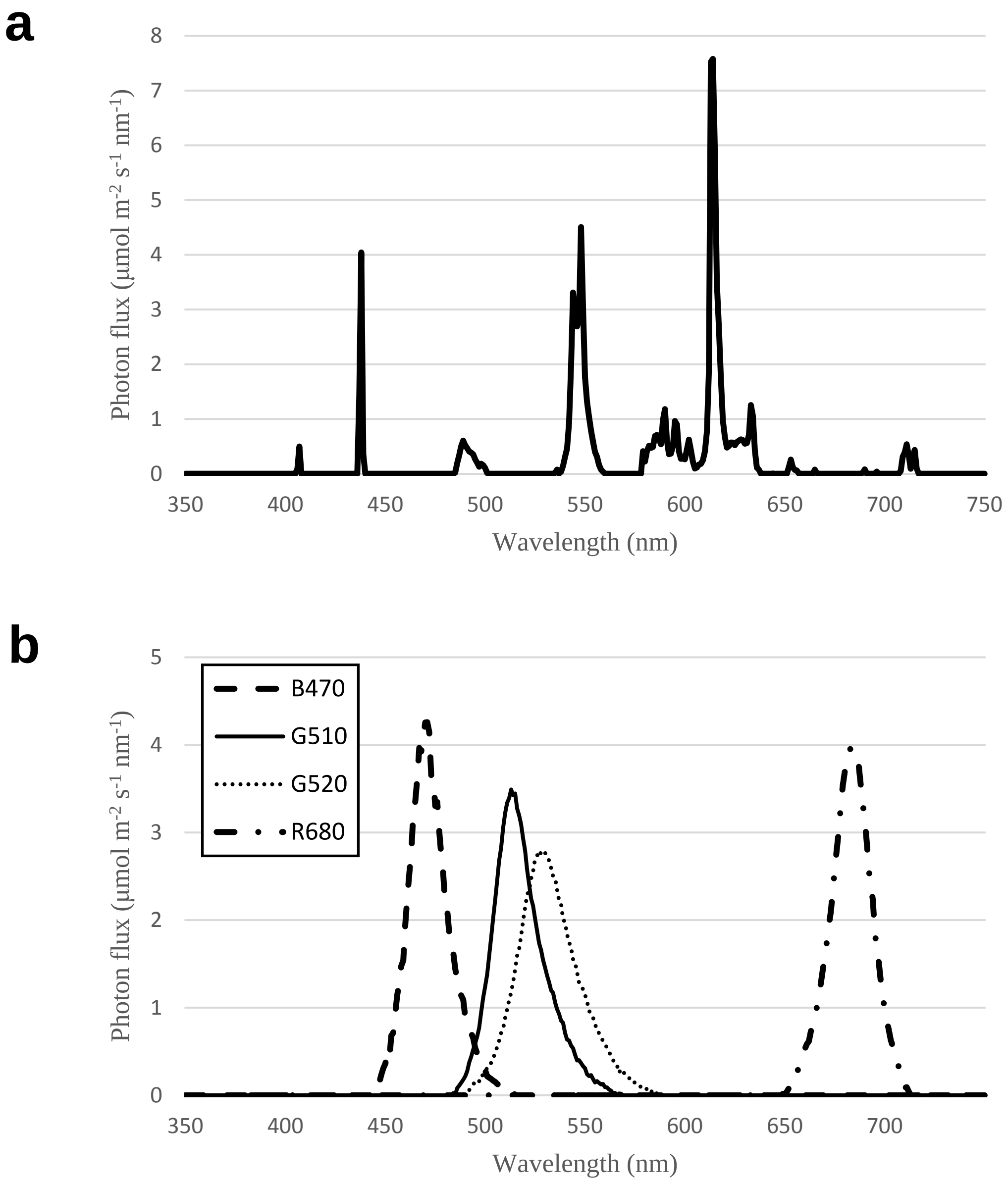
a
8
7
6
5
4
3
2
1
0
Photon flux (μmol m-2 s-1 nm-1)
350
400
450
500
550
600
650
700
750
Wavelength (nm)
b
5
4
3
2
1
0
B470
G510
G520
R680
Photon flux (μmol m-2 s-1 nm-1)
350
400
450
500
550
600
650
700
Wavelength (nm)

Figure S12

Supplementary Document S1. Metabolomics Metadata

1. Plant context metadata

1.1. Plant materials

1.1.1. BioSource Species

Lactuca sativa L.

1.1.2. Genotypes/Varieties

cv. Banchu Red Fire

1.1.3. Organ specification

The third leaf

1.1.4. Growth conditions

Under white fluorescent light (FL, FLR110H-W1A; Mitsubishi/Osram Co.; Yokohama, Japan), seeds of red-leaf lettuce (*Lactuca sativa* L. cv Banchu red fire; Takii seed, Kyoto, Japan) were pre-germinated [14 hr, 14 days, 23 ± 2ºC, 100 μmol m^{-2} s^{-1} photon synthetic photon flux density (PPFD)]. The seedlings were supplied with a nutrient solution (Otsuka hydroponic composition, Otsuka Chemical Co. Ltd., Osaka, Japan) adjusted to an electrical conductivity (EC) of 1.2 dS/m and pH 5.8. It contained 7.0 mmol l^{-1} NO_3^-, 0.6 mmol l^{-1} NH_4^+, 3.7 mmol l^{-1} K^+, 2.3 mmol l^{-1} Ca^{2+}, 1.3 mmol l^{-1} $H_2PO_4^-$, and 0.9 mmol l^{-1} Mg^{2+}. The seedlings were transplanted to cultivation panels in a growth chamber (VB1514; Vötsch, Germany), supplied with nutrient solution for the duration of the experiments, and grown at 25ºC [relative humidity (RH) 60%, 900 μmol mol^{-1} CO_2]. The plants were irradiated with different light spectra from LEDs, namely B470 (peak wavelength 470 nm, ISL-305X302-BBBB, CCS Co., Kyoto, Japan), G510 (peak wavelength 510 nm, ISL-305X302-GGGG505, CCS Co.), G520 (peak wavelength of 524 nm; ISL-305X302-GGGG525, CCS) and R680 (peak wavelength of 680nm; ISL-305X302-RRRR68, CCS Co., Kyoto, Japan) (Figure S9). The seedlings were irradiated for 24 hr at PPFD 100 or 300 μmol m^{-2} s^{-1} (P100, P300). The wavelength of the light source was determined with a USB2000 spectrometer (Ocean Optics, Dunedin, FL, USA) (Figure 1). At 14, 15 and 21 DAS, dry weight (DW) was measured.

2. Chemical analysis metadata

Chemicals

All the chemicals and reagents that were used for this study were of spectrometric grade. Chemicals excluding isotope reference compounds and reagents for silylation were purchased from Sigma Aldrich (Tokyo, Japan), NacalaiTesque (Kyoto, Japan), or Wako Pure Chemical Industries (Osaka, Japan). The 6 stable isotope compounds ([$^{13}C_5$]-proline, [$^{2}H_4$]-succinic acid, [$^{2}H_6$]-2-hydroxybenzoic acid, [$^{13}C_3$]-myristic acid, [$^{13}C_{12}$]-sucrose, and [$^{2}H_7$]-cholesterol) were purchased from Cambridge Isotope Laboratories (Andover, MA, USA); [$^{13}C_5$,^{15}N]-glutamic acid and [$^{13}C_6$]-glucose from Spectra Stable Isotopes (Columbia, Maryland, USA), [$^{2}H_4$]-1,4-diaminobutane was from C/D/N ISOTOPES (Pointe-Claire, Quebec, Canada), and [$^{13}C_4$]-hexadecanoic acid from Icon (Mt. Marion, NY, USA). The reagent for trimethylsilylation, *N*-methyl-*N*-trimethylsilyltrifluoroacetamide (MSTFA) was purchased from Tokyo Chemical Industry (Tokyo, Japan).

2.1. Sample processing and extraction

2.1.1. Extraction and derivatization for GC-TOF-MS

Each frozen sample with a 5-mm zirconia bead was extracted with 400 fold amount of solvent (methanol/chloroform/water [3:1:1 v/v/v]) containing 10 stable isotope reference compounds at 4°C in a mixer mill (MM301; Retsch, Haan, Germany) at a frequency of 15 Hz. Each isotope compound was adjusted to a final concentration of 15 ngper 1-μl injection volume. After 5-min centrifugation at 15,100 × g, a 200-μl aliquot of the supernatant was transferred to a glass insert vial. The extracts were evaporated to dryness in an SPD2010 SpeedVac® concentrator (Thermo Fisher, Scientific, Waltham, MA, USA). We used extracts from 0.5-mg DW samples for derivatization, i.e., methoxymation and silylation. For methoxymation, 30 μl of methoxyamine hydrochloride (20 mg/ml in pyridine) were added to the sample. After 22.5 h of derivatization at room temperature the sample was trimethylsilylated for 1 h using 30 µl of MSTFA at 37°C with shaking. All derivatization steps were performed in a vacuum glove box VSC-1000 (Sanplatec, Osaka, Japan) filled with 99.9995% (G3 grade) dry nitrogen.

2.1.2. Extraction for LC-q-TOF-MS to detect secondary metabolites

Each frozen sample was extracted with 5 fold amount of solvent (methanol/water [8:2 v/v]) containing a reference compound (2.5 μM of 10-camphorsulfonic acid ($[M-H]^-$, *m/z* 231.0691)) using

a mixer mill MM301 (Retsch) at a frequency of 18 Hz for 7 min at 4°C. After centrifugation for 10 min at 17,000 × g, the supernatant was filtered using an Oasis® HLB µElusion plate (30 µm, Waters Co., Massachusetts, US).

2.1.3. Extraction for LC-q-TOF-MS to detect lipids

Each frozen sample was milled using mixer mill MM301 (Retsch) at a frequency of 20 Hz for 2 min at 4°C. After that, frozen powder was extracted with 20 fold volume of extraction solvent (chloroform/methanol/water[50 : 100 : 31.45, v/v]) containing 1 µM of 1,2-didecanoyl-*sn*-glycero-3-phosphocholine (SIGMA). Samples were vigorously mixed using a vortex mixture. 52.6 µl of water and 52.6 µl of chloroform were added to 200 µl of extract and then vigorously mixed for 5 min at room temperature. After standing for 15 min on ice, the samples were centrifuged at 1,000 ×*g* at 5°C for 5 min. The lower layer (85 µl) was transferred to a 2 ml tube. Each extract was evaporated to dryness by SPD2010 SpeedVac® concentrator (Thermo Fisher Scientific). The residue was dissolved in 162 µl of ethanol, and centrifuged at 10,000×*g* at 5°C for 15 min. The supernatant was transferred to a glass insert and subjected to lipid analysis by LC-MS.

2.2. Analytical conditions

2.2.1. GC-TOF-MS conditions

Using the splitless mode of a CTC CombiPALautosampler (CTC Analytics, Zwingen, Switzerland), 1 µl of each sample (equivalent to 5.6 µg DW) was injected into an Agilent 6890N gas chromatograph (Agilent Technologies, Wilmingston, DE, USA) featuring a 30 m × 0.25 mm inner diameter fused-silica capillary column and a chemically bound 0.25-µl film Rxi-5 Sil MS stationary phase (RESTEK, Bellefonte, PA, USA) with a tandem connection to a fused silica tube (1 m, 0.15 mm). An MS column change interface (msNoVent-J; SGE, Yokohama, Japan) was used to prevent air and water from entering the MS during column change-over. Helium was the carrier gas at a constant flow rate of 1 ml min^{-1}. The temperature program for GC-MS analysis started with a 2-min isothermal step at 80°C followed by 30°C temperature-ramping to a final temperature of 320°C that was maintained for 3.5 min. The transfer line and the ion source temperatures were 250 and 200°C, respectively. Ions were generated by a 70-eV electron beam at an ionization current of 2.0 mA. The acceleration voltage was turned on after a solvent delay of 222 sec. Data acquisition was on a Pegasus IV TOF mass spectrometer (LECO, St. Joseph, MI, USA); the acquisition rate was 30 spectras^{-1} in the mass range of a mass-to-charge ratio of m/z = 60–800.

Alkane standard mixtures (C8 - C20 and C21 - C40) purchased from Sigma-Aldrich (Tokyo, Japan) were used for calculating the retention index (RI) [1]. For quality control we injected methylstearate into every 6th sample. The sample run order was randomized in single-sequence analyses. We analyzed the standard compound mixtures using the same sequence analysis procedures.

2.2.2. LC-q-TOF-MS conditions to detect secondary metabolites

After preparation of the extracts, the sample extracts (1 μl) were analyzed using an LC-MS system equipped with an electrospray ionization (ESI) interface (LC, Waters Acquity UPLC system; MS, Waters Xevo G2 Q-Tof). The analytical conditions were as follows. LC: column, Acquity bridged ethyl hybrid (BEH) C18 (pore size 1.7 μm, length 2.1× 100 mm, Waters); solvent system,acetonitrile(0.1% formic acid):water (0.1% formic acid); gradient program,

0.5 : 99.5 v/v at 0 min, 0.5 : 99.5 v/v at 0.1 min, 80 : 20 v/v at 10 min, 99.5 : 0.5 v/v at 10.1 min, 99.5 : 0.5 v/v at 12 min and 0.5 : 99.5 v/v at 12.1 min, 0.5 : 99.5 v/v at 15 min; flow rate, 0.3 ml/min; temperature, 40 °C; MS detection: capillary voltage, +3.0 keV, cone voltage, 25.0 V, source temperature, 120°C, desolvation temperature, 450 °C, cone gas flow, 50 l/ h; desolvation gas flow, 800 l/h; collision energy, 6 V; mass range, *m/z* 50−1500; scan duration, 0.1 sec; interscan delay, 0.014 sec; mode, centroid; polarity, negative; Lockspray (Leucineenkephalin): scan duration, 1.0 sec; interscan delay, 0.1 sec. The data were recorded using Progenesis CoMet (Nonlinear Dynamics).

2.2.3. LC-q-TOF-MS conditions to detect lipids

Sample extracts (1 μl) were analyzed using an LC-MS system equipped with an electrospray ionization (ESI) interface (HPLC, Waters Acquity UPLC system; MS, Waters Xevo G2 Qtof). Two-solvent (A and B) system was used for separation of each metabolite. Compositions of these solvents were as follows: solvent A, acetonitrile: water:1 M ammonium acetate:formic acid = (158 g:800g:10 ml:1 ml); solvent B, acetonitrile:2-propanol:water:1 M ammonium acetate:formic acid = (79 g:711 g:10 ml:1 ml). The analytical conditions were as follows. HPLC: column, Acquity UPLC HSS T3 (pore size 1.8 μm, 1.0 i.d × 50 mm long, Waters); gradient program, 35% B at 0 min, 70% B at 3 min, 85% B at 7 min, 90% B at 10 min, 90% B at 12 min and 35% B at 12.5 min; flow rate, 0.15 ml/min; temperature, 55°C; MS detection: capillary voltage, +3.0 kV; cone voltage, 20 V for positive mode and 40 V for negative mode; source temperature, 120°C; desolvation temperature, 450°C; cone gas flow, 50 l/h; desolvation gas flow, 450 l/h; collision energy, 6 V; detection mode, scan (*m/z* 100–

2000; scan time, 0. 5 sec; centroid). The scans were repeated for 15 min in a single run. The data were recorded using MassLynx version 4.1 software (Waters).

2.3. Data processing

2.3.1. Data processing for GC-TOF-MS data

Nonprocessed MS data from GC-TOF-MS analysis were exported in NetCDF format generated by chromatography processing- and mass spectral deconvolution software (LecoChromaTOF version 3.22; LECO, St. Joseph, MI, USA) to MATLAB 6.5 or MATLAB2011b (Mathworks, Natick, MA, USA) for the performance of all data-pretreatment procedures, e.g. smoothing, alignment, time-window setting H-MCR, and RDA [2]. The resolved MS spectra were matched against reference mass spectra using the NIST mass spectral search program for the NIST/EPA/NIH mass spectral library (version 2.0) and our custom software for peak-annotation written in JAVA. Peaks were identified or annotated based on their RIs, a comparison of the reference mass-spectra with the GolmMetabolome Database (GMD) released from CSB.DB [3], and our in-house spectral library. The metabolites were identified by comparison with RIs from the library databases (GMD and our own library) and the RIs of authentic standards. The metabolites were defined as annotated metabolites after comparison with the mass spectra and the RIs from these two libraries. The data matrix was normalized using the CCMN algorithm for further analysis [4] .

2.3.2. Data processing for LC-q-TOF-MS data to detect secondary metabolites

The data matrix was aligned by Progenesis CoMet (Nonlinear Dynamics).For normalization, intensity values of remained peaks was divided by those of the 10-camphorsulfonic acid ($[M\text{-}H]^-$, *m/z* 231.0691) after cutoff of the low-intensity peaks (less than 2000). Metabolite annotation was performed using a literature [5].

2.3.3. Data processing for LC-q-TOF-MS data to detect lipids

The data matrix was generated using the Makerlynx XS (Waters) using the profiling data files recorded in the MassLynx format (raw). The data matrices were processed using in-house Perl script. The original peak intensity values were divided with that of the internal standard (1,2-didecanoyl-*sn*-glycero-3-phosphocholine at *m/z* 566.382 $[M + H]^+$ and at *m/z* 610.372 $[M + HCOO]^-$ for the positive and negative ion modes, respectively) to normalize the peak intensity values among the metabolic profile data.

2.4. Statistical data analysis for metabolite profile data

The multi-platform data was summarized by unifying metabolite identifiers to a common referencing scheme using the MetMask tool [6]. The three matrices were then concatenated and correlated peaks with the same annotation were replaced by their first principal component. Principal component analysis (PCA) and orthogonal partial least square discriminant analysis (O2PLS-DA) were performed with $\log_{10}$ transformation and autoscaling using SIMCA-P 14.0 software (Umetrics AB, Umeå, Sweden).

References

1 Schauer, N. *et al.* GC-MS libraries for the rapid identification of metabolites in complex biological samples. *FEBS Lett* **579**, 1332-1337, doi:10.1016/j.febslet.2005.01.029 (2005).

2 Jonsson, P. *et al.* Predictive metabolite profiling applying hierarchical multivariate curve resolution to GC-MS data--a potential tool for multi-parametric diagnosis. *Journal of proteome research* **5**, 1407-1414, doi:10.1021/pr0600071 (2006).

3 Kopka, J. *et al.* GMD@CSB.DB: the Golm Metabolome Database. *Bioinformatics* **21**, 1635-1638, doi:10.1093/bioinformatics/bti236 (2005).

4 Redestig, H. *et al.* Compensation for systematic cross-contribution improves normalization of mass spectrometry based metabolomics data. *Anal Chem* **81**, 7974-7980, doi:10.1021/ac901143w (2009).

5 Abu-Reidah, I. M., Contreras, M. M., Arraez-Roman, D., Segura-Carretero, A. & Fernandez-Gutierrez, A. Reversed-phase ultra-high-performance liquid chromatography coupled to electrospray ionization-quadrupole-time-of-flight mass spectrometry as a powerful tool for metabolic profiling of vegetables: Lactuca sativa as an example of its application. *J Chromatogr A* **1313**, 212-227, doi:10.1016/j.chroma.2013.07.020 (2013).

6 Redestig, H. *et al.* Consolidating metabolite identifiers to enable contextual and multi-platform metabolomics data analysis. *BMC Bioinformatics* **11**, 214, doi:10.1186/1471-2105-11-214 (2010).
